# Identification of Novel Genetic Determinants of Erythrocyte Membrane Fatty Acid Composition among Greenlanders

**DOI:** 10.1371/journal.pgen.1006119

**Published:** 2016-06-24

**Authors:** Mette Korre Andersen, Emil Jørsboe, Camilla Helene Sandholt, Niels Grarup, Marit Eika Jørgensen, Nils Joakim Færgeman, Peter Bjerregaard, Oluf Pedersen, Ida Moltke, Torben Hansen, Anders Albrechtsen

**Affiliations:** 1 Section for Metabolic Genetics, The Novo Nordisk Foundation Center for Basic Metabolic Research, Faculty of Health and Medical Sciences, University of Copenhagen, Copenhagen, Denmark; 2 The Bioinformatics Centre, Department of Biology, University of Copenhagen, Copenhagen, Denmark; 3 Steno Diabetes Center, Gentofte, Denmark; 4 Villum Center for Bioanalytical Sciences, Department of Biochemistry and Molecular Biology, University of Southern Denmark, Odense, Denmark; 5 National Institute of Public Health, University of Southern Denmark, Copenhagen, Denmark; 6 Greenland Centre for Health Research, University of Greenland, Nuuk, Greenland; 7 Faculty of Health Sciences, University of Southern Denmark, Odense, Denmark; Wellcome Trust Sanger Institute, UNITED KINGDOM

## Abstract

Fatty acids (FAs) are involved in cellular processes important for normal body function, and perturbation of FA balance has been linked to metabolic disturbances, including type 2 diabetes. An individual’s level of FAs is affected by diet, lifestyle, and genetic variation. We aimed to improve the understanding of the mechanisms and pathways involved in regulation of FA tissue levels, by identifying genetic loci associated with inter-individual differences in erythrocyte membrane FA levels. We assessed the levels of 22 FAs in the phospholipid fraction of erythrocyte membranes from 2,626 Greenlanders in relation to single nucleotide polymorphisms genotyped on the MetaboChip or imputed. We identified six independent association signals. Novel loci were identified on chromosomes 5 and 11 showing strongest association with oleic acid (rs76430747 in *ACSL6*, beta (SE): -0.386% (0.034), p = 1.8x10^-28^) and docosahexaenoic acid (rs6035106 in *DTD1*, 0.137% (0.025), p = 6.4x10^-8^), respectively. For a missense variant (rs80356779) in *CPT1A*, we identified a number of novel FA associations, the strongest with 11-eicosenoic acid (0.473% (0.035), p = 2.6x10^-38^), and for variants in *FADS2* (rs174570), *LPCAT3* (rs2110073), and *CERS4* (rs11881630) we replicated known FA associations. Moreover, we observed metabolic implications of the *ACSL6* (rs76430747) and *CPT1A* (rs80356779) variants, which both were associated with altered HbA1c (0.051% (0.013), p = 5.6x10^-6^ and -0.034% (0.016), p = 3.1x10^-4^, respectively). The latter variant was also associated with reduced insulin resistance (HOMA-IR, -0.193 (0.050), p = 3.8x10^-6^), as well as measures of smaller body size, including weight (-2.676 kg (0.523), p = 2.4x10^-7^), lean mass (-1.200 kg (0.271), p = 1.7x10^-6^), height (-0.966 cm (0.230), p = 2.0x10^-5^), and BMI (-0.638 kg/m^2^ (0.181), p = 2.8x10^-4^). In conclusion, we have identified novel genetic determinants of FA composition in phospholipids in erythrocyte membranes, and have shown examples of links between genetic variants associated with altered FA membrane levels and changes in metabolic traits.

## Author Summary

Disruption of fatty-acid balance has in several previous studies been linked to human health conditions, including the metabolic syndrome, type 2 diabetes, and insulin resistance. Composition of fatty acids in lipid membranes is influenced, not only by diet and lifestyle, but also by genetic variation. By identifying genes linked to changes in the level of specific fatty acids, it may be possible to identify biological mechanisms and pathways central to regulation of fatty-acid composition in lipid membranes. We therefore aimed at finding such genes by studying Greenlanders. We identified six genomic regions harboring variants, which were associated with the level of at least one of 22 assessed erythrocyte membrane fatty acids, including two novel regions not previously linked to fatty acid levels. Moreover, we showed that two of the identified variants were associated with altered levels of glycosylated hemoglobin, and one of these variants was associated with reduced insulin resistance and decreased measures of body size. These results contribute to our understanding of fatty acid metabolism, and support a link between fatty acid balance and metabolic health.

## Introduction

Fatty acids (FAs) are important for normal body function, as they serve as essential structural entities of cellular membranes, as energy sources, and as signaling molecules. Perturbation of FA homeostasis may modulate membrane functions, cell signaling, and gene expression, and regulation of FA metabolism is, thus, critically important. Accordingly, variation in FA levels, measured in serum, plasma, erythrocyte membranes, or as dietary intake, has been linked to a range of cardiovascular [1–6] and metabolic risk factors, including the metabolic syndrome, dyslipidemia, type 2 diabetes, insulin resistance, and inflammation [7–10]. Moreover, significant correlation between erythrocyte membrane FAs and serum lipid levels has been observed [11–13], and this correlation has been suggested to be the link between erythrocyte membrane FA composition and metabolic diseases [13].

FAs are obtained from diet or synthesized endogenously from carbohydrate or protein sources by series of elongation and desaturation steps. ω-3 alpha-linolenic acid (18:3) and ω-6 linoleic acid (*cis-cis-*18:2) are essential FAs, which can only be obtained from diet and subsequently elongated and desaturated to form other ω-3 and ω-6 FAs. The levels of FAs in an individual are influenced by diet and lifestyle [14,15], but also have a clear hereditary component, which is estimated to account for 32–70% of FA variation [16,17]. The metabolic pathways regulating the circulating concentrations and membrane content of individual FAs, as well as the specific mechanisms linking FA levels to disease states are, however, poorly understood. We hypothesize, that improved understanding of these pathways and mechanisms can be achieved by identifying genetic loci associated with inter-individual differences in FA levels in erythrocyte membranes.

For genetic studies, the Greenlandic population is an important and powerful resource. This population has emerged from a historically small and isolated Inuit population, which has not until very recently admixed with Europeans [18]. Therefore, compared to European populations, the Greenlandic population has extended linkage disequilibrium (LD) and increased probability for presence of high frequency harmful variants due to genetic drift. These properties are advantageous in genetic studies, as they increase the statistical power to detect association signals. This was recently illustrated, when a novel type 2 diabetes associated variant, with an unusually high odds ratio of 10.3, was identified in a relatively small sample of Greenlanders [19]. The Greenlandic population also differs markedly from European populations with respect to diet. The traditional diet of the Greenlanders is rich in polyunsaturated ω-3 FAs derived mainly from marine mammals and fish [20], and has recently been shown to have had a large impact on genetic makeup through adaptive selection in Inuit [21].

In the present study, we aimed to identify novel genetic loci associated with inter-individual differences in the FA composition in erythrocyte membranes in a large-scale association study in Greenlanders.

## Results

The erythrocyte membrane FA-association analyses were conducted in 2,626 Greenlanders living in Greenland (57.5% women) from the population-based Greenlandic Inuit Health in Transition (IHIT) cohort. These individuals were on average 44.7 years old and had an average BMI of 26.4 kg/m^2^. We assessed the levels of 22 FAs in the phospholipid fraction of erythrocyte membranes, and analyzing MetaboChip genotyping data, we identified six independent association signals with a p-value under the significance threshold of 4.3x10^-7^ (S1 Fig). These six signals, mapped to genomic loci on chromosome 5, 11, 12, 19, and 20 (Table 1). For each of these regions we assessed imputed data for fine mapping, and assessed secondary FA associations as well as associations with metabolic phenotypes (S2 Fig). In the following, all associations are reported for the derived alleles in chromosomal order, and associations are reported down to the arbitrary p-value cut-off of p<1.0x10^-3^.

**Table 1:** Summary of MetaboChip association signals with p-values <4.3x10^-7^.

Chr	Lead SNP	In gene	Derived allele (DAF)	Fatty acid	p-value
5	rs251015	*FNIP1*	C (0.692)	Oleic acid (18:1 ω-9)	2.5x10^-17^
11	rs174570	*FADS2*	T (0.783)	Adrenic acid (22:4 ω-6)	7.3x10^-19^
11	rs1551304	*TPCN2*	T (0.180)	Lignoceric acid (24:0)	1.6x10^-17^
12	rs2110073	*PHB2*	C (0.872)	Oleic acid (18:1 ω-9)	1.1x10^-9^
19	rs2913968	*RAB11B*	T (0.570)	Aarachidic acid (20:0)	2.7x10^-7^
20	rs6035106	*DTD1*	C (0.577)	Docosahexaenoic acid (22:6 ω-3)	6.4x10^-8^

Lead SNP refers to the SNP with the lowest p-value in the MetaboChip data.

Chr, chromosome; DAF, derived allele frequency.

**Fig 1:**
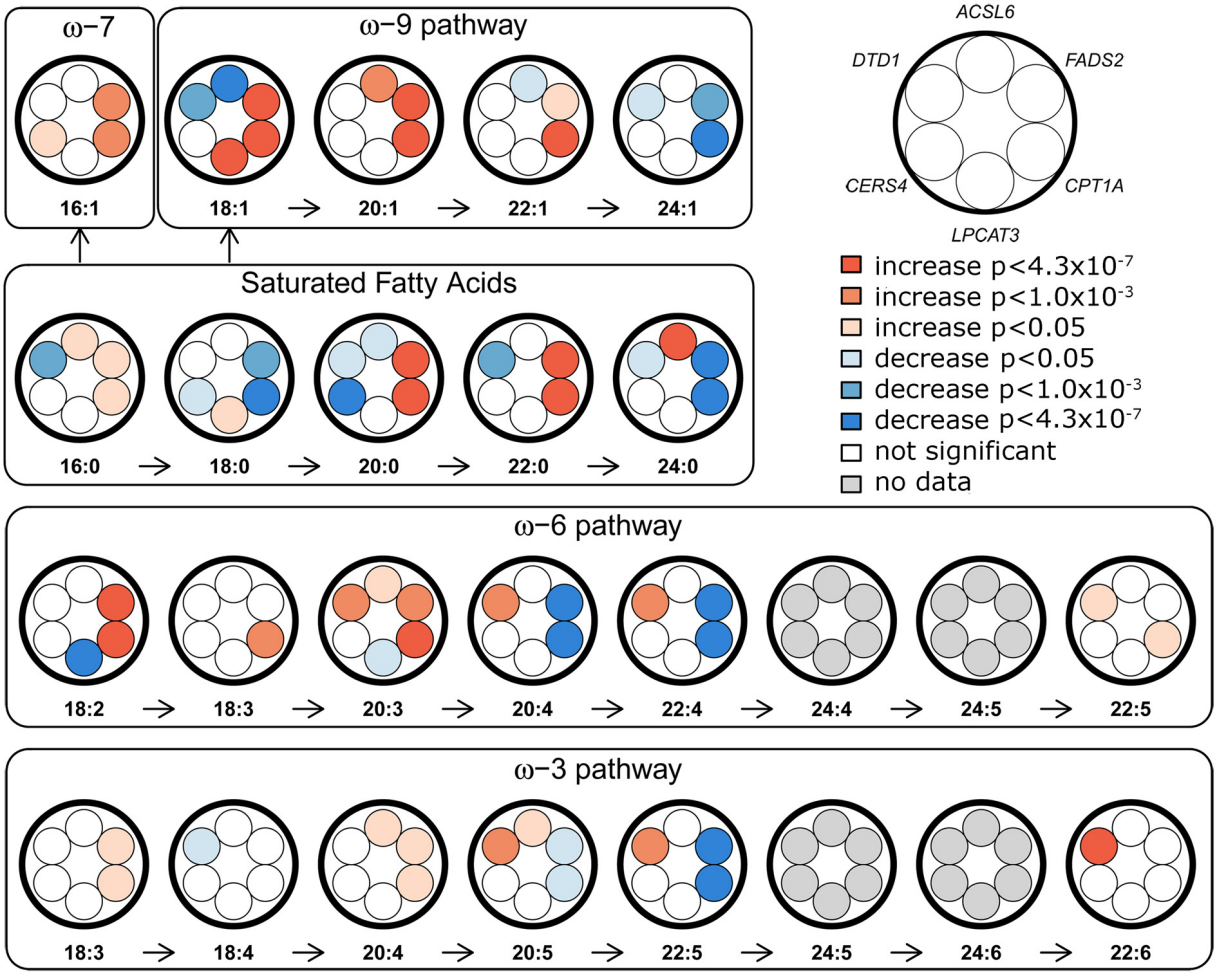
Overview of FA-synthesis pathways and candidate-SNP associations. Effects of candidate SNPs from the six identified erythrocyte membrane FA-associated loci are shown for each of the 22 assessed FAs. *ACSL6*, rs76430747; *FADS2*, rs174570; *CPT1A*, rs80356779; *LPCAT3*, rs2110073; *CERS4*, rs11881630; *DTD1*, rs6035106.

**Fig 2:**
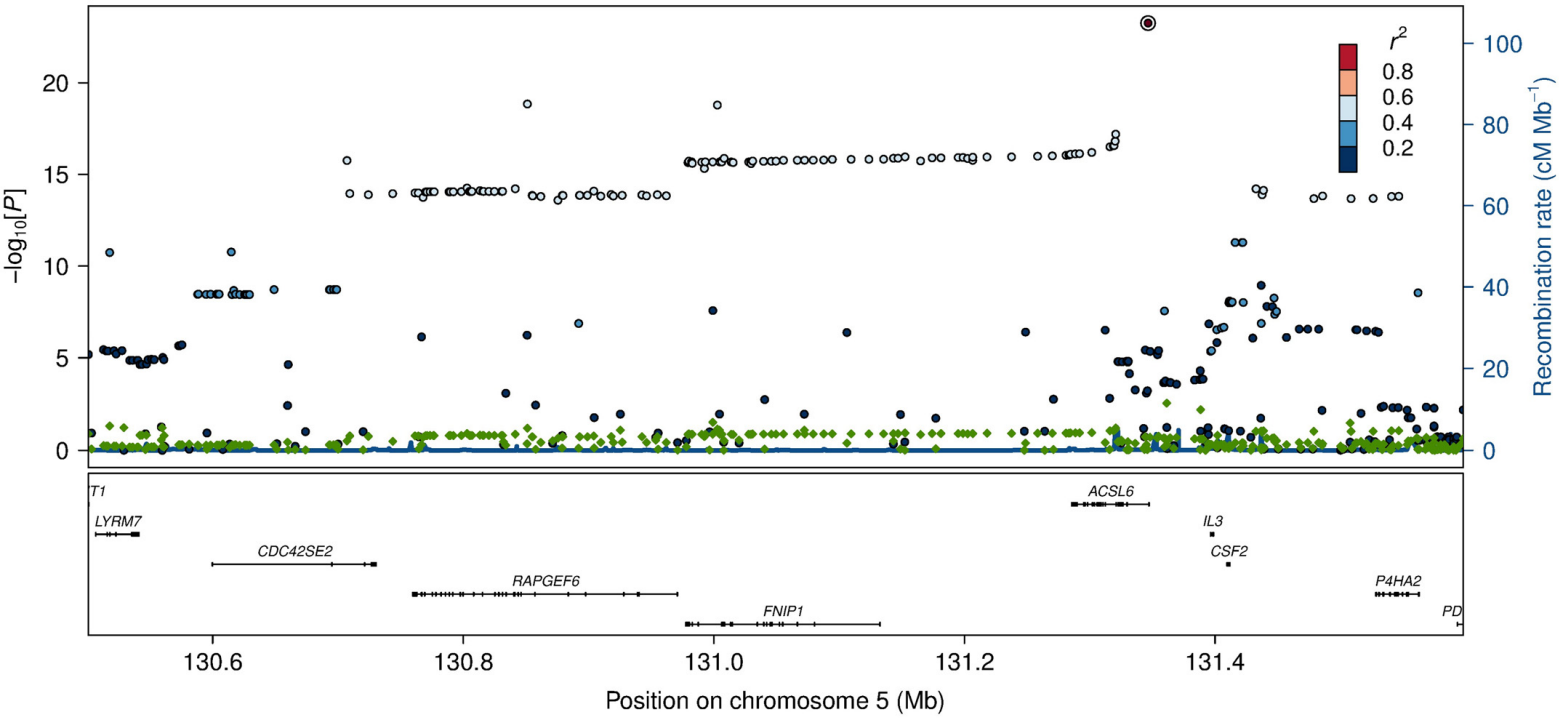
Association and conditional plot of the *ACSL6* locus with erythrocyte membrane oleic acid (18:1 ω-9). The association results of the unconditional analysis are colored according to the LD, which is calculated for the candidate SNP in the region, rs76430747. Green dots represent the results of the conditional analysis, and the circle denotes the SNP conditioned on (rs76430747). The p-values are based on imputation data.

**Fig 3:**
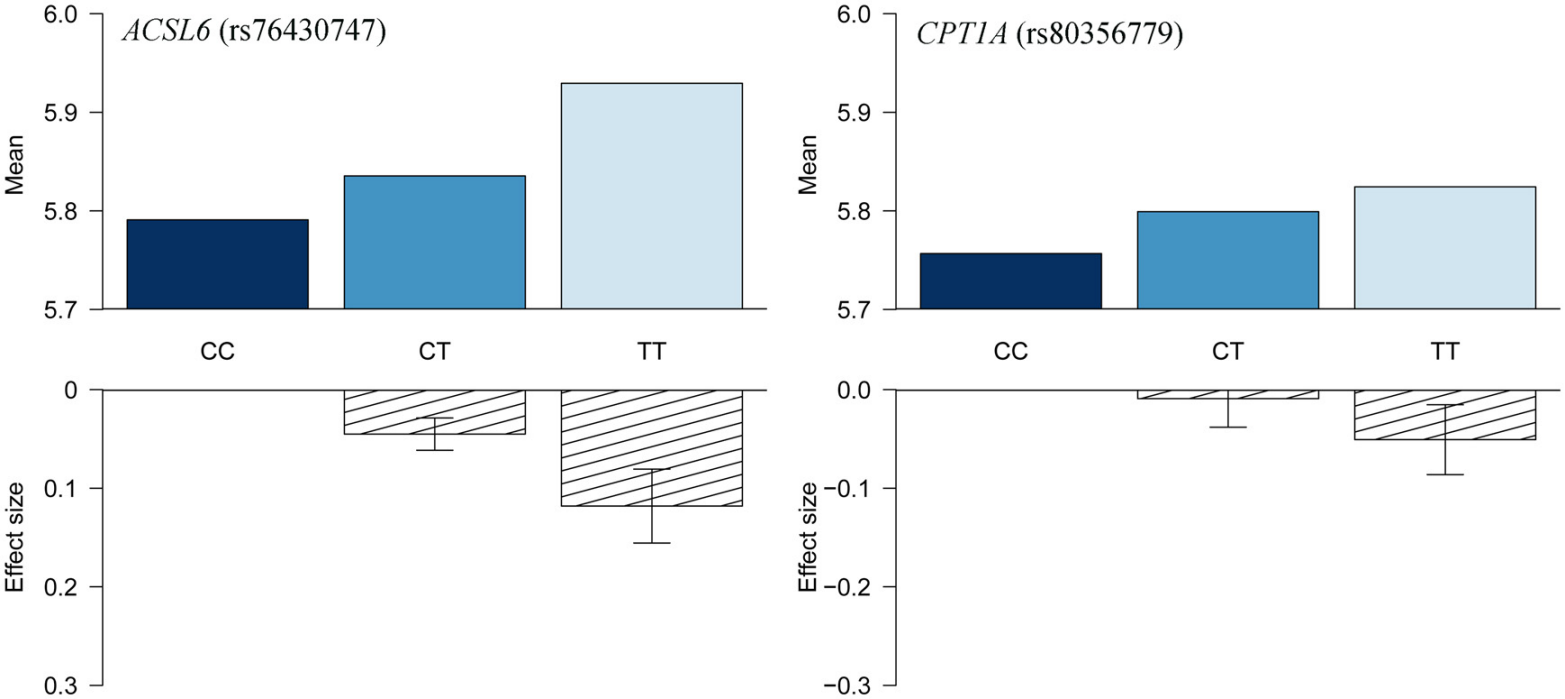
HbA1c association for *ACSL6* (rs76430747) and *CPT1A* (rs80356779). Data are shown as raw means stratified by genotype, and as effect sizes estimated without assuming an additive effect model.

The first association signal mapped to a novel locus on **chromosome 5**. The lead SNP (rs251015) was located in intronic region in *FNIP1*, and the derived allele showed strongest association with higher levels of oleic acid (18:1 ω-9). The imputation data revealed another SNP in the locus, rs76430747, located in an intronic region of *ACSL6*. The derived allele of rs76430747 was associated with lower levels of oleic acid (18:1 ω-9), and higher levels of lignoceric acid (24:0) and 11-eicosenoic acid (20:1 ω-9; Table 2 and Fig 1). Conditional analyses showed that rs76430747 may explain the observed association with oleic acid (Fig 2), whereas the results for lignoceric acid and 11-eicosenoic acid were less clear (S3 Fig). Interestingly, the derived allele of rs76430747 was, in addition to FA levels, associated with higher HbA1c (Fig 3 and Table 3).

**Table 2:** Effect sizes for candidate-SNP associations with fatty acids.

Chromosome	5	11	11	12	19	20
Candidate gene	*ACSL6*	*FADS2*	*CPT1A*	*LPCAT3*	*CERS4*	*DTD1*
Candidate SNP	rs76430747	rs174570	rs80356779	rs2110073	rs11881630	rs6035106
Derived allele (DAF)	T (0.180)	T (0.783)	T (0.730)	C (0.872)	T (0.200)	C (0.577)
ω-3 pathway						
Eicosapentaenoic acid (20:5)						0.081 (0.021)*
Docosapentaenoic acid (22:5)		-0.268 (0.046)**	-0.379 (0.042)**			0.120 (0.026)*
Docosahexaenoic acid (22:6)						0.137 (0.025)**
ω-6 pathway						
Linoleic acid (*cis-cis*-18:2)		0.316 (0.037)**	0.394 (0.034)**	-0.207 (0.034)**		
Gamma-linolenic acid (18:3)			0.036 (0.010)*			
Dihomo-gamma-linolenic acid (20:3)		0.185 (0.039)*	0.332 (0.035)**			0.082 (0.023)*
Arachidonic acid (20:4)		-0.328 (0.045)**	-0.319 (0.040)**			0.109 (0.025)*
Adrenic acid (22:4)		-0.375 (0.041)**	-0.455 (0.036)**			0.083 (0.023)*
ω-7 pathway						
Palmitoleic acid (16:1)		0.198 (0.046)*	0.206 (0.042)*			
ω-9 pathway						
Oleic acid (18:1)	-0.386 (0.034)**	0.322 (0.048)**	0.400 (0.043)**	0.256 (0.042)**		-0.098 (0.028)*
11-eicosenoic acid (20:1)	0.132 (0.029)*	0.348 (0.039)**	0.473 (0.035)**			
Erucic acid (22:1)			0.098 (0.017)**			
Nervonic acid (24:1)		-0.150 (0.042)*	-0.294 (0.038)**			
Saturated FAs						
Palmitic acid (16:0)						-0.116 (0.027)*
Stearic acid (18:0)		-0.164 (0.048)*	-0.228 (0.044)**			
Arachidic acid (20:0)		0.296 (0.047)**	0.408 (0.043)**		-0.225 (0.033)**	
Behenic acid (22:0)		0.331 (0.040)**	0.436 (0.037)**			-0.086 (0.025)*
Lignoceric acid (24:0)	0.178 (0.031)**	-0.230 (0.043)**	-0.410 (0.038)**			

Effect sizes are displayed as beta (standard error), and were estimated by transforming the FA values to a standard normal distribution, except for 18:3 ω-6 and 22:1 ω-9, where transformation was binary.

Candidate SNP refers to the best causal candidate SNP in each locus. The effect size with the lowest p-value for each SNP is marked with bold.

* denotes p<1.0x10^-3^

** denotes p<4.3x10^-7^.

DAF, derived allele frequency.

**Table 3:** Candidate-SNP associations with metabolic traits.

Chr	SNP	Candidate gene	Derived allele (DAF)	Trait	Beta (SE)	p-value
5	rs76430747	*ACSL6*	T (0.180)	HbA1c (%)	0.051 (0.013)	5.6x10^-6^
11	rs80356779	*CPT1A*	T (0.730)	Weight (kg)	-2.676 (0.523)	2.4x10^-7^
				Lean mass (kg)	-1.200 (0.271)	1.7x10^-6^
				HOMA-IR	-0.193 (0.050)	3.8x10^-6^
				Fs-insulin (pmol/l)	-5.710 (1.535)	5.9x10^-6^
				Height (cm)	-0.966 (0.230)	2.0x10^-5^
				Hip (cm)	-1.386 (0.333)	2.4x10^-5^
				BMI (kg/m^2^)	-0.638 (0.181)	2.8x10^-4^
				HbA1c (%)	-0.034 (0.016)	3.1x10^-4^
				Waist (cm)	-1.693 (0.464)	5.1x10^-4^

Untransformed effect sizes for metabolic traits reaching p<1.0x10^-3^.

SNP refers to the best causal candidate in the locus.

Chr, chromosome; DAF, derived allele frequency; SE, standard error.

The second and the third association signals mapped to **chromosome 11**. The strongest signal from the MetaboChip data (rs174570) was located in intronic sequence of the well-established FA-associated *FADS2*. rs174570 showed association with the level of 13 FAs (Table 2). The second signal on chromosome 11, mapped a little more than 7Mb away from the *FADS* region to intronic sequence of *TPCN2*. Similar to the *FADS2* variant, the MetaboChip lead SNP in this locus (rs1551304) showed association with a large number of FAs (S4 Table). Imputation based analyses revealed no better candidate SNPs in the region, instead we genotyped a known missense variant (rs80356779) in the nearby candidate gene *CPT1A*, attempting to identify the causal variant for this second locus.

The *CPT1A* rs80356779 variant accentuated the association pattern across all assessed FA pathways observed for rs1551304, however, in the opposite direction for the derived allele, but the same direction for the minor alleles. Also, it mirrored the association pattern observed for the rs174570 *FADS2* variant (Fig 1, Table 2). Specifically, the *CPT1A* rs80356779 showed a pattern where the levels of ω-7 palmitoleic acid (16:1) was increased, the downstream products of the ω-3 (docosapentaenoic acid, 22:5), ω-6 (arachidonic acid, 20:4 and adrenic acid, 22:4), ω-9 (nervonic acid, 24:1), and saturated fatty acid (SFA; lignoceric acid, 24:0) pathways were reduced, and the levels of the corresponding upstream precursors (ω-6, linoleic acid (*cis-cis-*18:2), gamma-linolenic acid (18:3), and dihomo-gamma-linolenic acid (20:3); ω-9, oleic acid (18:1), 11-eicosenoic acid (20:1), and erucic acid (22:1); SFA, arachidic acid (20:0) and behenic acid (22:0)) were increased (Fig 1 and Table 2). In addition to the FA associations, *CPT1A* rs80356779 was associated with lower HbA1c (Fig 3), indicating improved glycemic regulation, and with several measures of smaller body size and reduced insulin resistance (Table 3).

**Fig 4:**
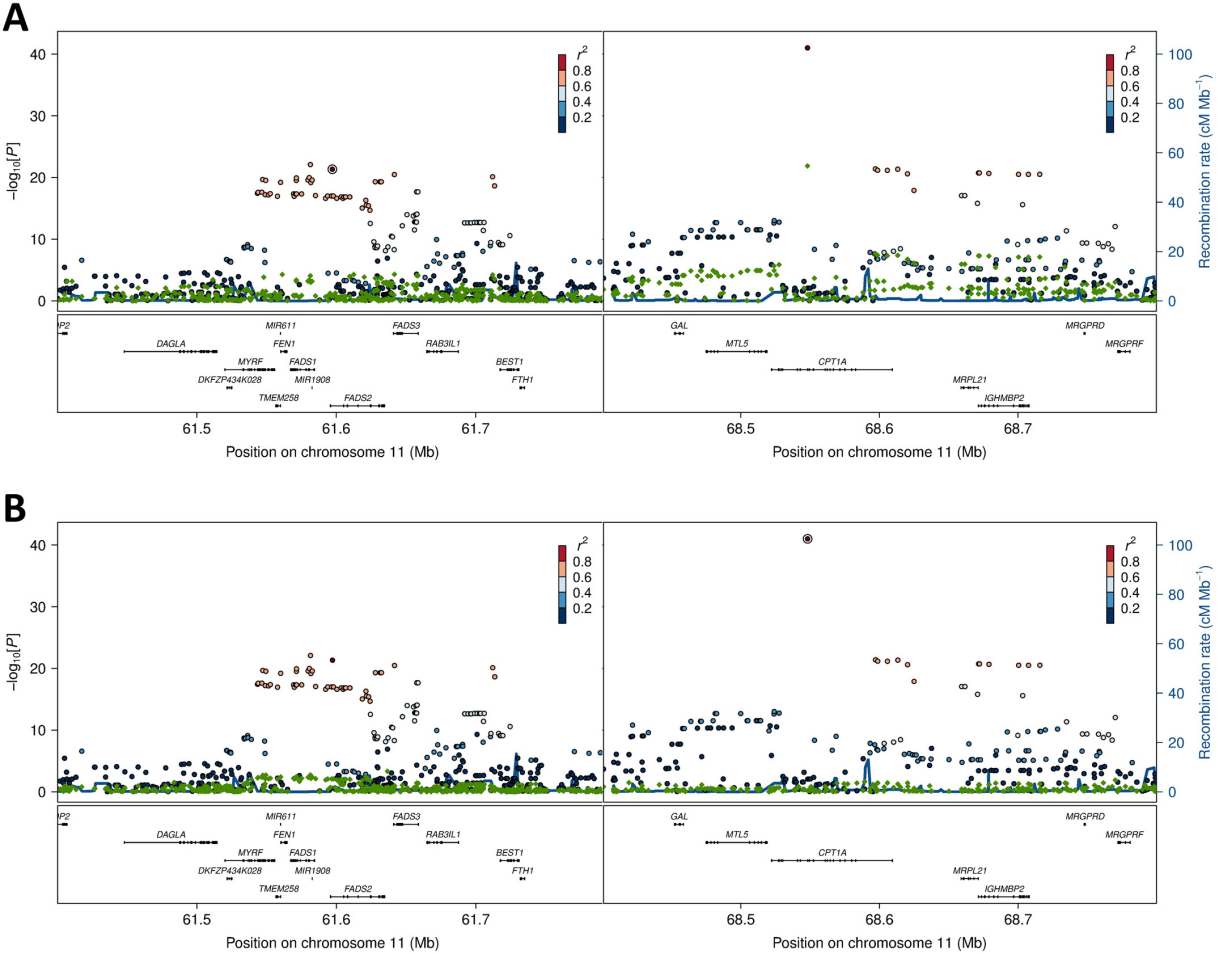
Association and conditional plot combined for the *FADS2* and *CPT1A* loci with erythrocyte membrane 11-eicosenoic acid (20:1 ω-9). The association results of the unconditional analysis are colored according to the LD, which was calculated separately for the two regions in each plot for the candidate SNPs, rs174570 and rs80356779, respectively. The p-values are based on imputation data. Green dots represent the results of the conditional analyses, and the circle denotes the SNP conditioned on A) rs174570 and B) rs80356779.

Further exploration of the region revealed an unusual LD phenomenon, where *FADS2* rs174570 and *CPT1A* rs80356779 were linked in the ancestral Inuit population despite the chromosomal distance of approximately 7 Mb. Both variants were fixed or at extremely low frequency for the derived allele in the ancestral Inuit population, while the ancestral allele of rs80356779 was entirely fixed, and the ancestral allele of rs174570 was very common in European populations (Table 4). Conditional analysis reduced the association signal for both loci, as seen for 11-eicosenoic acid (20:1 ω-9; Fig 4). In general, most of the *CTP1A* associations were still significant when conditioning on the *FADS2* variant, while many of the *FADS2* associations disappeared when conditioning on the *FADS2* variant (Fig 4 and S4 Fig).

**Table 4:** *FADS2-CPT1A* linkage pattern.

Population	*FADS2-CPT1A* haplotype frequency estimates				r^2^
	C-C	C-T	T-C	T-T	
Inuit	0	0	0.007	0.993	0
European	0.686	0.067	0.236	0.012	0.004
Greenland	0.200	0.018	0.072	0.711	0.588

Estimates are based on the admixture proportions determined by [18], and are given for the ancestral components (Inuit and European), as well as the Greenlandic population.

The fourth association signal mapped to **chromosome 12**, where the MetaboChip lead SNP (rs2110073) was located in intronic sequence in *PHB2*. This variant was associated with higher levels of oleic acid (18:1 ω-9), and lower levels of linoleic acid (*cis-cis*-18:2 ω-6; Fig 1 and Table 2). Imputation based analyses revealed additional SNPs in the region in high LD with rs2110073 mapping to nearby genes including the known FA-associated *LPCAT3* (S5 Fig). However, none of these SNPs had a significantly better p-value than rs2110073, and were therefore not analyzed further. Conditional analyses indicated that rs2110073 may explain the associations observed for oleic acid (18:1 ω-9) and linoleic acid (*cis-cis*-18:2 ω-6; S5 Fig)

The fifth association signal mapped to **chromosome 19**. The lead SNP (rs2913968) was located in intronic region of *RAB11B*, and was associated with the SFA arachidic acid (20:0). Imputation analyses pointed to another possible causal SNP, rs11881630, mapping to *CERS4*. Additional genotyping of this synonymous exome variant showed a stronger association signal with decreased levels of arachidic acid than observed for rs2913968 (Fig 1 and Table 2). However, association analyses conditioning on rs2913968 and rs11881630, respectively, were inconclusive in determining which, if any, of the variants that caused the association signal (S6 Fig), and with the available data it was impossible to exclude the possibility of two independent association signals.

The sixth and final association signal mapped to a novel locus on **chromosome 20**, where the MetaboChip lead SNP (rs6035106) was located in an intronic region of *DTD1*. The derived allele of this SNP was associated with higher levels of docosahexaenoic acid (22:6 ω-3), and in addition showed an interesting pattern of associations, with generally higher ω-3 and ω-6 FA levels, but lower SFA levels (Fig 1 and Table 2). The imputation data revealed no candidate SNPs in the region with a significantly better p-value, and the conditional analysis on rs6035106 showed that this variant may explain the observed docosahexaenoic acid (22:6 ω-3) association, as well as the other FA associations (S7 Fig).

## Discussion

We identified six distinct genomic regions harboring variants significantly associated with the level of at least one of 22 assessed erythrocyte membrane FAs. These genomic regions comprise two novel loci not previously linked to FA levels (*ACSL6*, *DTD1*), one known FA-linked locus where we identify a broad range of novel FA associations (*CPT1A*), and three loci for which we replicate known FA-associations (*FADS2*, *LPCAT3*, *CERS4*).

The first of the two novel FA-associated loci (rs76430747) is located on in *ACSL6* chromosome 5. The ACSL6 protein is a long-chain acyl-CoA synthase that activates FAs by catalyzing the formation of acyl-CoA from ATP, CoA, and FAs [22]. This step is required for FAs to enter most metabolic pathways including protein modification, phospholipid synthesis, and beta-oxidation. ACSL proteins, thus, affect the distribution and amount of intracellular FAs, and the isoforms differ in tissue specificity and FA preference [23]. ACSL6 is mainly expressed in brain and neuronal cells, and catalyzes very-long chain FAs, containing 18–26 carbons [24]. Our results may indicate that rs76430747 causes a shift in the preference for FA incorporated into phospholipids in cellular membranes towards ω-9 11-eicosenoic acid (20:1) and the SFA lignoceric acid (24:0), as opposed to ω-9 oleic acid (18:1), which seems to be directed to degradation by beta-oxidation. Alternatively, ACSL6 facilitated activation of oleic acid (18:1) to its corresponding acyl-CoA may channel this type of FA to elongation by ELOVL3 [25], explaining the observed increase in 11-eicosenoic acid (20:1) levels. Interestingly, ACSL6 has been shown to activate oleic acid in rat cell [26,27], which supports our functional hypothesis.

Besides from their role as intermediates in lipid synthesis and FA degradation, saturated and unsaturated long-chain acyl-CoAs have also been linked to regulation of carbohydrate and lipid metabolism, gene expression, and insulin secretion [28,29]. These functional properties may explain the observed association between variation in *ACSL6* and increased HbA1c. However, this association was not supported by differences in measures of insulin secretion or glucose levels, and requires replication to be validated.

The second novel FA-associated locus we identified is located on chromosome 20. The lead SNP (rs6035106) showed an interesting pattern of altered FA concentrations, with higher levels of ω-3 and ω-6 FAs, and reduced levels of SFAs, however, only associated with modest p-values. Based on this pattern we hypothesize that the causal variant in this locus induces a shift in the choice of FAs absorbed from diet, or in the specificity towards FAs incorporated into erythrocyte membrane lipids. The observed association signals mapped to *DTD1*, however, the product of this gene has no known functional link to FA-metabolic pathways [30]. The gene-rich region contains other possible causal candidate genes, among these are *SCP2D1*. This gene is particularly interesting, as it encodes a protein functionally related to the protein product of *SCP2* [31–33]. The SCP2 protein has been proposed to redistribute numerous lipid species, including FAs and fatty-acyl CoAs between lipid rafts and intracellular organelles, and thus regulate signaling pathways [31]. Additional studies are needed to validate the putative association between this genomic region and FA levels, and to determine if *SCP2D1* is the causal gene in the region.

On chromosome 11 we identified a locus showing a broad range of novel FA associations. The lead SNP in this region is a missense variant (rs80356779) in *CPT1A*, located around 7 Mb from the *FADS*-gene region. The *FADS* genes have been shown to be associated with a range of FAs primarily in European populations in candidate gene studies [34–38], and in large-scale genetic studies [39–44], and these associations were replicated in this study. The *CPT1A* rs80356779 variant causes a proline to leucine substitution at position 479 in the CPT1A protein sequence (P479L), and has been studied previously in Arctic populations [45–47]. Conditional analyses indicate that *CPT1A* P479L is the causal variant in the region among Inuit. However, the *FADS* locus must also harbor a true association signal, as such a signal has been observed in many studies in European populations [46–48], where P479L is monomorphic. The *FADS* and *CPT1A* association signals are difficult to untangle, because rs174570 and rs80356779 are both fixed in the ancestral Inuit population. This unusual long-range LD phenomenon is mainly due to recent adaptive selection on these loci, and lack of admixture from other populations until only a couple of hundred years ago [21,49].

*CPT1A* encodes a member of the carnitine palmitoyltransferase (CPT) protein family. These proteins catalyzes the initial and rate-limiting step in mitochondrial degradation of FAs in skeletal muscle, liver, and adipose tissue [50], and are thus attractive candidates to affect the levels of individual FAs. The CPT1A isoform is mainly expressed in liver [51], and cell-line studies and animal models have indicated that the P479L variant results in increased CPT1A activity, and thereby possibly increased flow of FAs to degradation by beta-oxidation [52,53]. We hypothesize, that increased CPT1A activity is the functional explanation for the observed changes in FA concentrations across the ω-3, ω-6, ω-7, ω-9, and SFA pathways. This extensive association pattern is a novel observation. Previously only association with the ω-6 FAs linoleic acid (*cis-cis-*18:2), dihomo-gamma-linolenic acid (20:3), and arachidonic acid (20:4) have been reported for three *CPT1A* intron variants [54]. Our results indicate that the Inuit specific P479L is the causal variant in *CPT1A* explaining the observed FA associations.

Moreover, *CPT1A* P479L T-allele carriers (L479) showed reduced insulin resistance and smaller body size. This phenotypic impact of L479 is an extension of the previously reported association with reduced body fat and central obesity in Yup’ik Eskimos [47], and it is well in line with data from functional studies showing that increased FA oxidation is linked to reduced accumulation of body fat and improved lipid profile [55–58], whereas inhibition of fat oxidation by a CPT1 inhibitor in rodents causes increased adiposity and insulin resistance [59]. We also observed association between L479 and improved glycemic regulation, which may simply be a consequence of the genotypic effect on body size and insulin resistance. In the Yup’ik Eskimos as well as in Greenlandic Inuit L479 has been associated with higher circulating levels of HDL cholesterol [46,47], this was not replicated in our study despite our larger sample size. The genomic region containing *CPT1A* seems to have been under increased evolutionary pressure, and to be of particular importance in Arctic populations [48,49]. This may be due to the traditional Greenlandic diet, which is enriched with animal fat, accentuating the requirement for FA beta-oxidation facilitated by CPT1A for generating energy.

In addition to the novel associations and the well-established *FADS2* association discussed above we also replicated the association between rs2110073, near the functional candidate gene *LPCAT3* on chromosome 12, and altered levels of the first precursors for endogenous synthesis of the ω-6 (linoleic acid, *cis-cis-*18:2) and ω-9 (oleic acid, 18:1) FAs [44], and the association between variation in *CERS4* and levels of arachidic acid (20:0) [60–62]. The originally reported *CERS4* SNP, rs2100944 [60], barely reached nominal significance in our study (p = 0.013), instead we identified another causal candidate SNP in this locus, namely rs11881630. This variant is rare in Europeans (MAF: 0.89%), and is in low LD with rs2100944 both in Europeans and in Greenlanders, thus, indicating that neither of the two is the causal variant, or that there may be two association signals.

### Conclusions and perspectives

We have identified six independent loci associated with erythrocyte membrane FA levels in Greenlanders. The Greenlandic population is characterized by geographical isolation, and has a unique genetic profile shaped by genetic drift due to both founder events and a small population size through thousands of years. Moreover, the Greenlanders have adapted their lifestyle to cold climate and available resources, resulting in a highly specialized diet rich in fat and protein from fish and marine mammals [20]. This extreme Greenlandic lifestyle has resulted in genetic adaptation, demonstrated by identification of positive selection in the *FADS* genes on chromosome 11 [21]. The importance of this region in adaption to Arctic lifestyle is further supported by positive selection on *CPT1A* in Siberians [49]. Besides from the selection signatures in the *FADS* and *CPT1A* loci, we observed no indications of selection acting on the FA-associated regions in our data. Thus, based on our data it seems unlikely that the unique FA composition of the traditional Greenlandic diet has inferred selective pressure on the *ACSL6*, *LPCAT3*, *CERS4*, and *DTD1* gene regions.

In conclusion, we have identified genetic determinants of FA composition of erythrocyte membrane phospholipids possibly as a result of altered fluxes through FA and lipid metabolic pathways. These alterations may have metabolic consequences, supported by the observed link between FA associated variants and altered glycemic regulation and body composition. The present study provides a framework to further delineate how specific lipid species regulate human metabolic disorders.

## Materials and Methods

### Ethics statement

The study was approved by the Commission for Scientific Research in Greenland (project 2011–13, ref. no. 2011–056978; and project 2013–13, ref.no. 2013–090702), and was conducted in accordance with the ethical standards of the Declaration of Helsinki, second revision. All participants gave informed consent.

### Study population

The study included individuals from three Greenlandic cohorts: the IHIT (n = 3,115) and B99 (n = 1,401) cohorts, which comprise Greenlanders living in Greenland, and the BBH (n = 547) cohort, which comprises Greenlanders living in Denmark. The B99 and the IHIT cohorts were collected as part of a general population health survey of the Greenlandic population during 1999–2001 and 2005–2010, respectively, as described in [63,64]. BBH was collected in Denmark during 1998–1999 from people of Greenlandic descent [64]. There was an overlap of individuals between IHIT and B99, these individuals (n = 295) were assigned to the B99 cohort.

### Measurements and assays

Fatty acids (FAs) in phospholipids of erythrocytes were measured in 2,626 individuals from the IHIT cohort of Greenlanders living in Greenland. FA levels were reported as relative levels compared to the total amount of FAs in each sample. Lipids were separated by thin layer chromatography, after extraction of total lipids with chloroform/methanol, and FAs in the phospholipid fraction were methylated, and subsequently analyzed by capillary GLC using a HP-Packard GC chromatograph equipped with a DB-23 column at The Centre de recherche sur les maladies lipidiques (CRML), Centre hospitalier universitaire de Québec. We restricted the genetic analyses to 22 FAs for which we observed detectable levels for the majority of individuals, and which could be placed in the FA synthesis pathways (Fig 1). These FAs comprised ω-3 (18:3, alpha-linolenic acid; 18:4, stearidonic acid; 20:4, eicosatetraenoic acid; 20:5, eicosapentaenoic acid; 22:5, docosapentaenoic acid; 22:6, docosahexaenoic acid), ω-6 (*cis-cis*-18:2, linoleic acid; 18:3, gamma-linolenic acid; 20:3, dihomo-gamma-linolenic acid; 20:4, arachidonic acid; 22:4, adrenic acid; 22:5, docosapentaenoic acid), ω-7 (16:1, palmitoleic acid), ω-9 (18:1, oleic acid; 20:1, 11-eicosenoic acid; 22:1, erucic acid; 24:1, nervonic acid), and SFAs (16:0, palmitic acid; 18:0, stearic acid; 20:0, arachidic acid; 22:0, behenic acid; 24:0, lignoceric acid).

All IHIT and B99 participants above 35 years of age underwent an oral glucose tolerance test, where blood samples were drawn after an overnight fast of at least 8 hours, and 2 hours after receiving a 75 g glucose bolus. Plasma glucose levels were analyzed with the Hitachi 912 system (Roche Diagnostics), serum insulin with an immunoassay excluding des-31,32 split products and intact proinsulin (AutoDELFIA, PerkinElmer), and Hba1c by ion-exchange HPLC (G7, Tosoh Bioscience). Insulin resistance was estimated by the homeostasis model assessment (HOMA-IR), calculated as [(fasting glucose level x fasting insulin level)/6.945]/22.5, where insulin levels were expressed as pmol/l and glucose levels as mmol/l [65]. Information about diet was obtained from questionnaires, as described in [66].

### Genotyping

DNA was purified from blood leukocytes, and all samples were genotyped on the Metabochip (Illumina). This chip is a custom iSelect genotyping array of 196,725 single nucleotide polymorphisms (SNPs) for genetic studies of metabolic, cardiovascular, and anthropometric traits [67]. Genotyping was performed using the HiScan system (Illumina), and genotypes were called jointly for all cohorts using the GenCall module of the GenomeStudio software (Illumina) using default cluster data. The data set went through a two-step quality control. In step one, duplicate samples and individuals missing >2% genotypes or with gender discrepancy were removed. In step two, we removed SNPs with a minor allele frequency <1% (<5% for case-control transformed phenotypes), with >100 missing genotypes, with a large deviation from Hardy Weinberg equilibrium (p<1.0x10^-10^), as well as SNPs which were polymorphic in the IHIT cohort but not in the B99+BBH cohorts, and SNPs associated with gender (p<1.0x10^-5^). In total, 4,674 individuals (2,791 from IHIT, 1,336 from B99, and 547 from BBH) and 115,182 SNPs passed the quality control.

MetaboChip data was used to identify loci harboring association signals (p<4.3x10^-7^, corresponding to correction for testing 115,182 SNPs). Based on these data and imputation analyses of the identified loci, three additional SNPs were selected for genotyping (rs76430747, rs11881630, and rs11028474). Moreover, based on previous reports of association with lipid and body composition traits in the Alaskan Yup’ik Eskimos [47] and high frequency in Canadian and Greenlandic Inuit [45,46] an additional candidate SNP in the *CPT1A* locus (rs80356779), was also selected for genotyping (LGC genomics). Analyzing genotype data against MetaboChip data for rs11028474 showed discrepancy, hence, this variant was removed from all analyses.

### Imputation

In order to fine map loci identified to harbor an association signal in the MetaboChip data, we carried out imputation to increase the number of SNPs in these regions. For the imputation we applied Omni5M array (Illumina) genotype data from 20 Greenlandic trios, which was phased using ShapeIt [68]. The phased data of the 40 Greenlandic parents and 1000 genomes data for 41 Europeans and 40 Han Chinese were applied as reference panel. IMPUTE2 [69] was used for imputation, where a recombination map for the reference SNPs was inferred with linear interpolation using the hg19 genomic map from IMPUTE2 as a template, and an effective population size of 1,500 [21]. The imputation generated genotype data (R^2^ >0.4) for 1,959,225 variants, which were analyzed as dosages (mean genotype) using GEMMA with the same setup as for the other statistical analyses using a relatedness matrix based on the dosages instead of genotypes, see below.

### Statistical analysis

For association tests we applied a linear mixed model, implemented in the software GEMMA [70], to account for relatedness and admixture. For each phenotype the tests were applied to data from all individuals across the three cohorts with information about that specific phenotype, and the relatedness matrix required as input to GEMMA was estimated from genotypic data from these individuals only. For all tests we assumed an additive effect and included sex, age, and cohort as covariates, and for tests involving FAs we included dietary intake of marine mammals, and percentage of the diet consisting of traditional Inuit food as additional covariates. The FA association analyses were also run without the adjustment for diet, and with additional adjustment for the use of lipid-lowering drugs to exclude confounding from these factors. These adjustments did not alter the results, but are included in the supplementary tables for the FA-associated variants for comparison (S1–S10 Tables).

Prior to performing association tests, quantitative traits were quantile transformed to a standard normal distribution within each sex. However, six FAs (alpha-linolenic acid (18:3 ω-3), stearidonic acid (18:4 ω-3), eicosatetraenoic acid (20:4 ω-3), gamma-linolenic acid (18:3 ω-6), docosapentaenoic acid (22:5 ω-6), and erucic acid (22:1 ω-9)) had undetectable levels in more than 20% of the samples. We, thus, considered transformation to a standard normal distribution inappropriate. Instead these traits were transformed to case-control like data, by transforming values different from 0 to 1, and they were analyzed as detectable levels (cases) and undetectable levels (controls).

In analyses of the MetaboChip data, we applied a significance threshold of p<4.3x10^-7^, corresponding to correction for testing 115,182 SNPs, to identify loci harboring association signals. These loci were selected for further analyses, including imputation efforts, and assessment of secondary FA associations and metabolic phenotypes (S1–S10 Tables and S2 Fig). All associations are described for the derived alleles, and reported down to the arbitrary p-value cut-off of p<1.0x10^-3^.

## Supporting Information

S1 Fig Manhattan and QQ plots from analyses of MetaboChip data, for each of the 22 assessed erythrocyte membrane FAs. The dashed line in the Manhattan plots denotes the significance threshold of p<4.3x10^-7^. P-values are calculated based on data transformed either to a standard normal or binary distribution. A) alpha-linolenic acid (18:3 ω-3), B) stearidonic acid (18:4 ω-3), C) eicosatetraenoic acid (20:4 ω-3), D) eicosapentaenoic acid (20:5 ω-3), E) docosapentaenoic acid (22:5 ω-3), F) docosahexaenoic acid (22:6 ω-3), G) linoleic acid (*cis-cis*-18:2 ω-6), H) gamma-linolenic acid (18:3 ω-6), I) dihomo-gamma-linolenic acid (20:3 ω-6), J) arachidonic acid (20:4 ω-6), K) adrenic acid (22:4 ω-6), L) docosapentaenoic acid (22:5 ω-6), M) palmitoleic acid (16:1 ω-7), N) oleic acid (18:1 ω-9), O) 11-eicosenoic acid (20:1 ω-9), P) erucic acid (22:1 ω-9), Q) nervonic acid (24:1 ω-9), R) palmitic acid (16:0), S) stearic acid (18:0), T) arachidic acid (20:0), U) behenic acid (22:0), and V) lignoceric acid (24:0). The rs11028474 variant on chromosome 11 causes the strong but spurious association signals for eicosapentaenoic acid (20:5 ω-3), docosapentaenoic acid (22:5 ω-3), docosahexaenoic acid (22:6 ω-3), and stearic acid (18:0). This variant was removed from all analyzes (see Materials and Methods section).
(PDF)

S2 Fig Flow chart of the study. (PDF)

S3 Fig Association and conditional plots. Unconditional and conditional association analyses for rs76430747 in the ACSL6 locus with A) 11-eicosenoic acid (20:1 ω-9) and B) lignoceric acid (24:0).The association results of the unconditional analysis are colored according to the LD, which is calculated for the candidate SNP in the region. Green dots represent the results of the conditional analysis, and the circles denote the SNPs conditioned on. The p-values are based on imputation data.
(PDF)

S4 Fig Association and conditional plots. Unconditional and conditional association analyses for rs174570 and rs80356779 in the FADS2 and CPT1A loci, respectively, with A) palmitoleic acid (16:1 ω-7), B) stearic acid (18:0), C) oleic acid (18:1 ω-9), D) linoleic acid (cis-cis-18:2 ω-6), E) gamma-linolenic acid (18:3 ω-6), F) arachidic acid (20:0), G) 11-eicosenoic acid (20:1 ω-9), H) dihomo-gamma-linolenic acid (20:3 ω-6), I) arachidonic acid (20:4 ω-6), J) behenic acid (22:0), K) erucic acid (22:1 ω-9), L) adrenic acid (22:4 ω-6), M) docosapentaenoic acid (22:5 ω-3), N) lignoceric acid (24:0), and O) nervonic acid (24:1 ω-9).The association results of the unconditional analysis are colored according to the LD, which is calculated for the candidate SNP in the region. Green dots represent the results of the conditional analysis, and the circles denote the SNPs conditioned on. The p-values are based on imputation data.
(PDF)

S5 Fig Association and conditional plots. Unconditional and conditional association analyses for rs2110073 in the LPCAT3 locus with A) oleic acid (18:1 ω-9) and B) linoleic acid (cis-cis-18:2 ω-6).The association results of the unconditional analysis are colored according to the LD, which is calculated for the candidate SNP in the region. Green dots represent the results of the conditional analysis, and the circles denote the SNPs conditioned on. The p-values are based on imputation data.
(PDF)

S6 Fig Association and conditional plots. Unconditional and conditional association analyses for A) rs2913968 and B) rs11881630 in the CERS4 locus with arachidic acid (20:0).The association results of the unconditional analysis are colored according to the LD, which is calculated for the candidate SNP in the region. Green dots represent the results of the conditional analysis, and the circles denote the SNPs conditioned on. The p-values are based on imputation data.
(PDF)

S7 Fig Association and conditional plots. Unconditional and conditional association analyses for rs6035106 in the DTD1 locus with A) eicosapentaenoic acid (20:5 ω-3), B) docosapentaenoic acid (22:5 ω-3), C) docosahexaenoic acid (22:6 ω-3), D) dihomo-gamma-linolenic acid (20:3 ω-6), E) arachidonic acid (20:4 ω-6), F) adrenic acid (22:4 ω-6), G) oleic acid (18:1 ω-9), H) palmitic acid (16:0), and I) behenic acid (22:0).The association results of the unconditional analysis are colored according to the LD, which is calculated for the candidate SNP in the region. Green dots represent the results of the conditional analysis, and the circles denote the SNPs conditioned on. The p-values are based on imputation data.
(PDF)

S1 Table Association results for rs251015 (*FNIP1*). (XLSX)

S2 Table Association results for rs76430747 (*ACSL6*). (XLSX)

S3 Table Association results for rs174570 (*FADS2*). (XLSX)

S4 Table Association results for rs1551304 (*TPCN2*). (XLSX)

S5 Table Association results for rs80356779 (*CPT1A*). (XLSX)

S6 Table Association results for rs2110073 (*PHB2*). (XLSX)

S7 Table Association results for rs2913968 (*RAB11B*). (XLSX)

S8 Table Association results for rs11881630 (*CERS4*). (XLSX)

S9 Table Association results for rs6035106 (*DTD1*). (XLSX)

S10 Table Information about SNPs of interest. (XLSX)
