# Supplementary material for: Identification of Novel Genetic Determinants of Erythrocyte Membrane Fatty Acid Composition among Greenlanders
Source: PLoS Genet. 2016 Jun 24;12(6):e1006119. doi: 10.1371/journal.pgen.1006119 (PMC4920407; doi:10.1371/journal.pgen.1006119)
Supplement: S1 Fig — The dashed line in the Manhattan plots denotes the significance threshold of p<4.3x10^-7^. P-values are calculated based on data transformed either to a standard normal or binary distribution. A) alpha-linolenic acid (18:3 ω-3), B) stearidonic acid (18:4 ω-3), C) eicosatetraenoic acid (20:4 ω-3), D) eicosapentaenoic acid (20:5 ω-3), E) docosapentaenoic acid (22:5 ω-3), F) docosahexaenoic acid (22:6 ω-3), G) linoleic acid (cis-cis-18:2 ω-6), H) gamma-linolenic acid (18:3 ω-6), I) dihomo-gamma-linolenic acid (20:3 ω-6), J) arachidonic acid (20:4 ω-6), K) adrenic acid (22:4 ω-6), L) docosapentaenoic acid (22:5 ω-6), M) palmitoleic acid (16:1 ω-7), N) oleic acid (18:1 ω-9), O) 11-eicosenoic acid (20:1 ω-9), P) erucic acid (22:1 ω-9), Q) nervonic acid (24:1 ω-9), R) palmitic acid (16:0), S) stearic acid (18:0), T) arachidic acid (20:0), U) behenic acid (22:0), and V) lignoceric acid (24:0). The rs11028474 variant on chromosome 11 causes the strong but spurious association signals for eicosapentaenoic acid (20:5 ω-3), docosapentaenoic acid (22:5 ω-3), docosahexaenoic acid (22:6 ω-3), and stearic acid (18:0). This variant was removed from all analyzes (see Materials and Methods section). (PDF) [file pgen.1006119.s001.pdf]

S1 Fig. Manhattan and QQ plots from analyses of MetaboChip data, for each of the 22 assessed erythrocyte membrane FAs. The dashed line in the Manhattan plots denotes the significance threshold of $p<4.3x10^{-7}$. P-values are calculated based on data transformed either to a standard normal or binary distribution. A) alpha-linolenic acid (18:3 ω-3), B) stearidonic acid (18:4 ω-3), C) eicosatetraenoic acid (20:4 ω-3), D) eicosapentaenoic acid (20:5 ω-3), E) docosapentaenoic acid (22:5 ω-3), F) docosahexaenoic acid (22:6 ω-3), G) linoleic acid (*cis-cis*-18:2 ω-6), H) gamma-linolenic acid (18:3 ω-6), I) dihomo-gamma-linolenic acid (20:3 ω-6), J) arachidonic acid (20:4 ω-6), K) adrenic acid (22:4 ω-6), L) docosapentaenoic acid (22:5 ω-6), M) palmitoleic acid (16:1 ω-7), N) oleic acid (18:1 ω-9), O) 11-eicosenoic acid (20:1 ω-9), P) erucic acid (22:1 ω-9), Q) nervonic acid (24:1 ω-9), R) palmitic acid (16:0), S) stearic acid (18:0), T) arachidic acid (20:0), U) behenic acid (22:0), and V) lignoceric acid (24:0). The rs11028474 variant on chromosome 11 causes the strong but spurious association signals for eicosapentaenoic acid (20:5 ω-3), docosapentaenoic acid (22:5 ω-3), docosahexaenoic acid (22:6 ω-3), and stearic acid (18:0). This variant was removed from all analyzes (see Materials and Methods section).

A

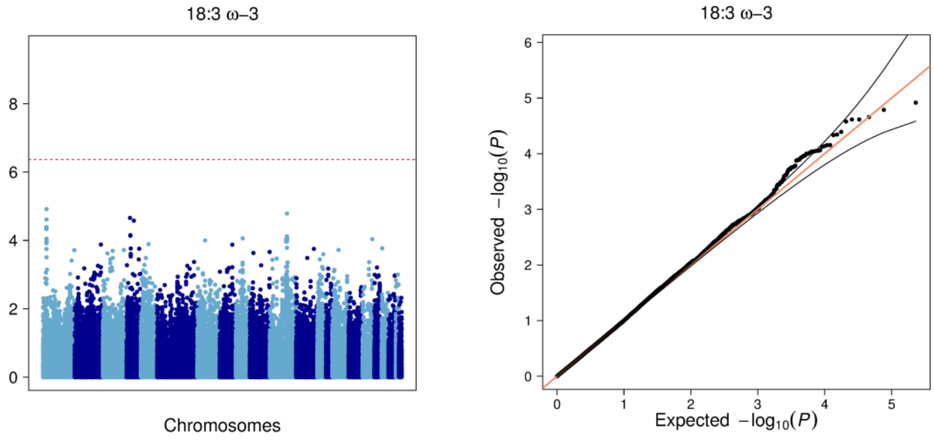

B

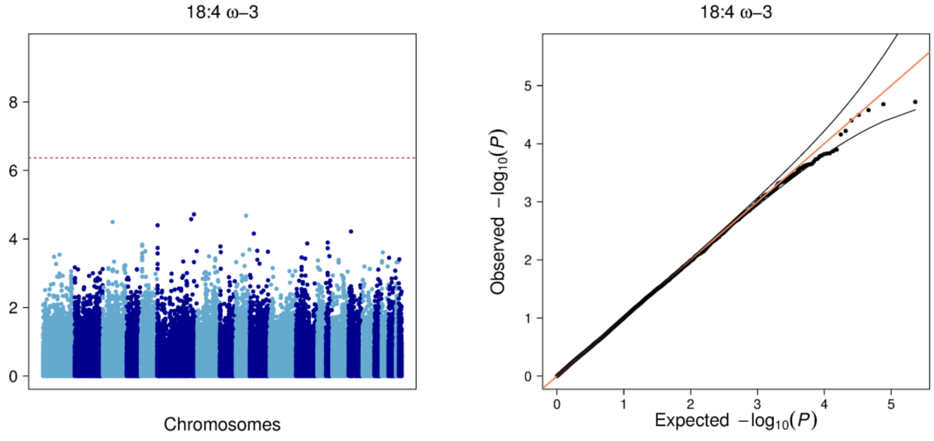

C

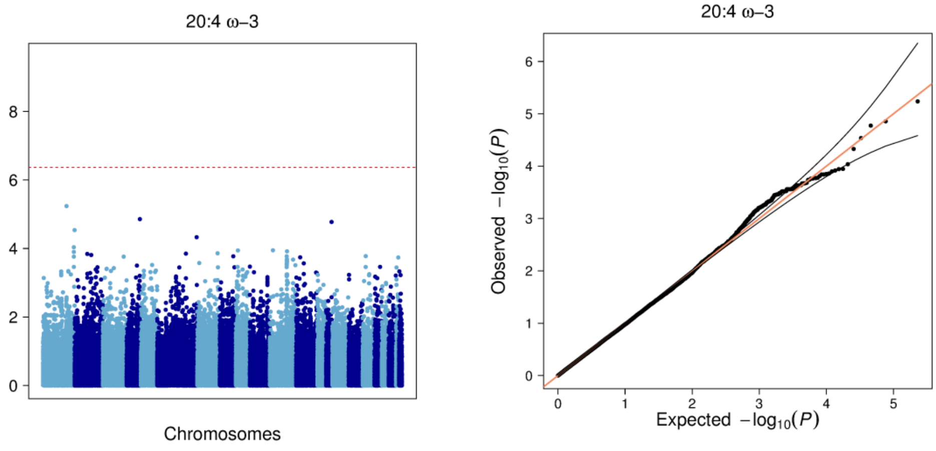

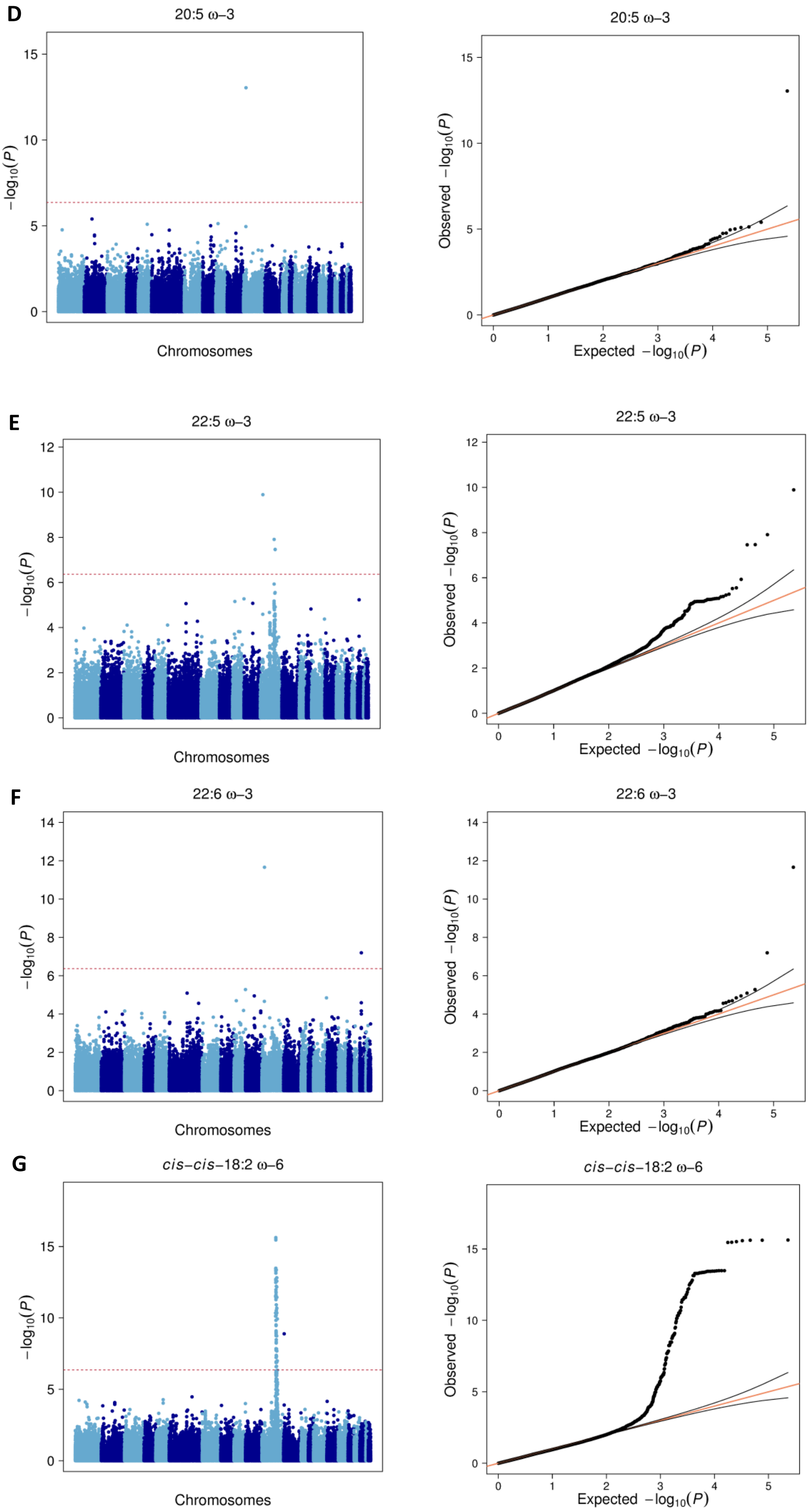
D
20:5 ω−3
−log10(P)
Chromosomes
20:5 ω−3
Observed −log10(P)
Expected −log10(P)
E
22:5 ω−3
−log10(P)
Chromosomes
22:5 ω−3
Observed −log10(P)
Expected −log10(P)
F
22:6 ω−3
−log10(P)
Chromosomes
22:6 ω−3
Observed −log10(P)
Expected −log10(P)
G
cis−cis−18:2 ω−6
−log10(P)
Chromosomes
cis−cis−18:2 ω−6
Observed −log10(P)
Expected −log10(P)

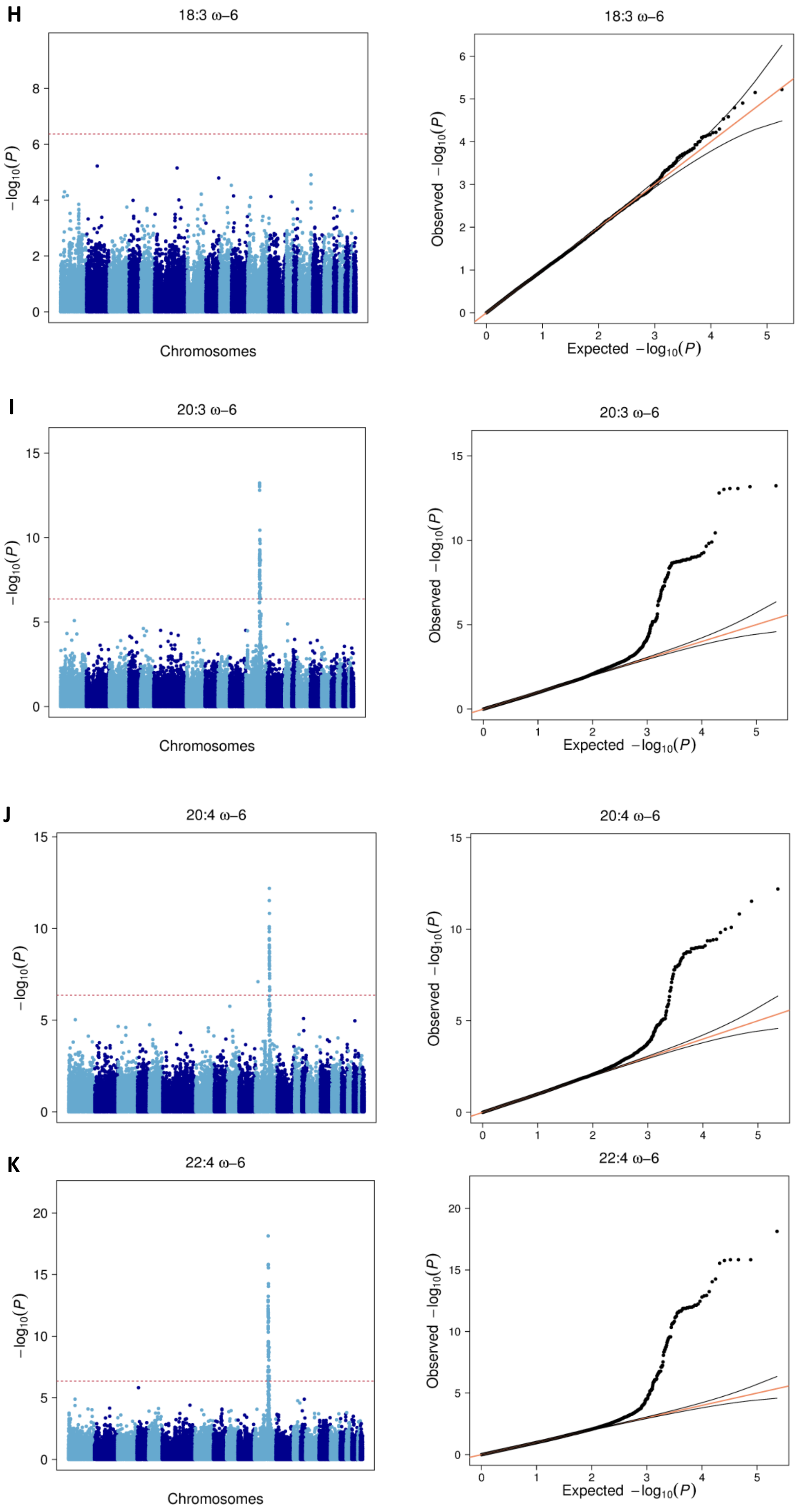
H
18:3 ω–6
$-\log_{10}(P)$
Chromosomes
18:3 ω–6
Observed $-\log_{10}(P)$
Expected $-\log_{10}(P)$
I
20:3 ω–6
$-\log_{10}(P)$
Chromosomes
20:3 ω–6
Observed $-\log_{10}(P)$
Expected $-\log_{10}(P)$
J
20:4 ω–6
$-\log_{10}(P)$
20:4 ω–6
Observed $-\log_{10}(P)$
K
22:4 ω–6
$-\log_{10}(P)$
Chromosomes
22:4 ω–6
Observed $-\log_{10}(P)$
Expected $-\log_{10}(P)$

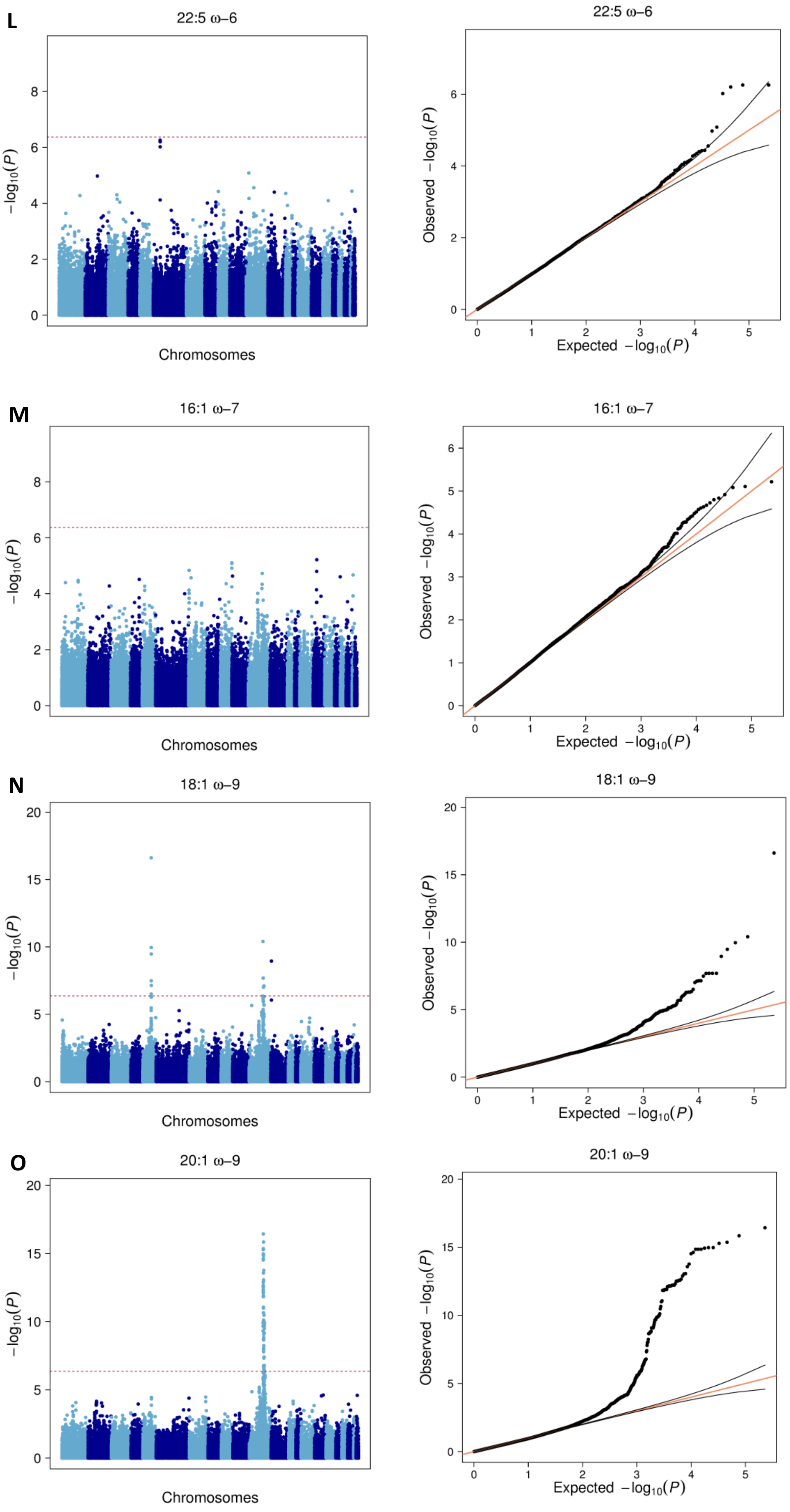
L
22:5 ω–6
−log10(P)
Chromosomes
Observed −log10(P)
Expected −log10(P)
M
16:1 ω–7
−log10(P)
Chromosomes
Observed −log10(P)
Expected −log10(P)
N
18:1 ω–9
−log10(P)
Chromosomes
Observed −log10(P)
Expected −log10(P)
O
20:1 ω–9
−log10(P)
Chromosomes
Observed −log10(P)
Expected −log10(P)

P

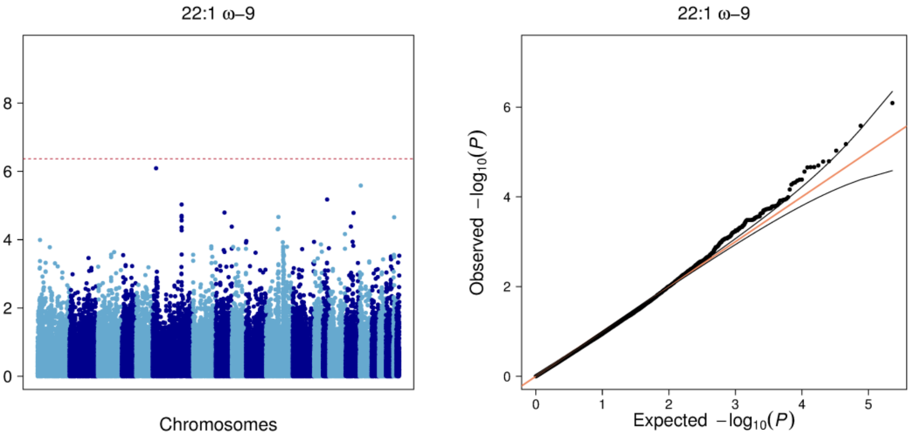
22:1 ω–9
−log10(P)
Chromosomes
22:1 ω–9
Observed −log10(P)
Expected −log10(P)

Q

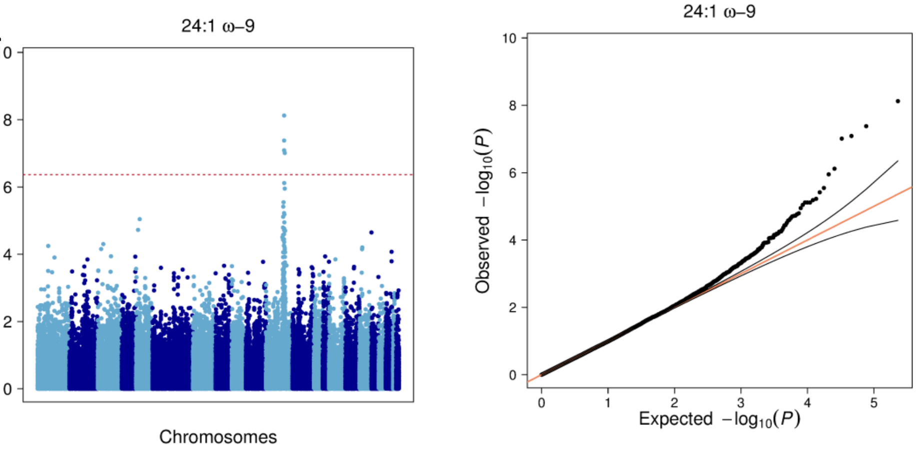
24:1 ω–9
−log10(P)
Chromosomes
24:1 ω–9
Observed −log10(P)
Expected −log10(P)

R

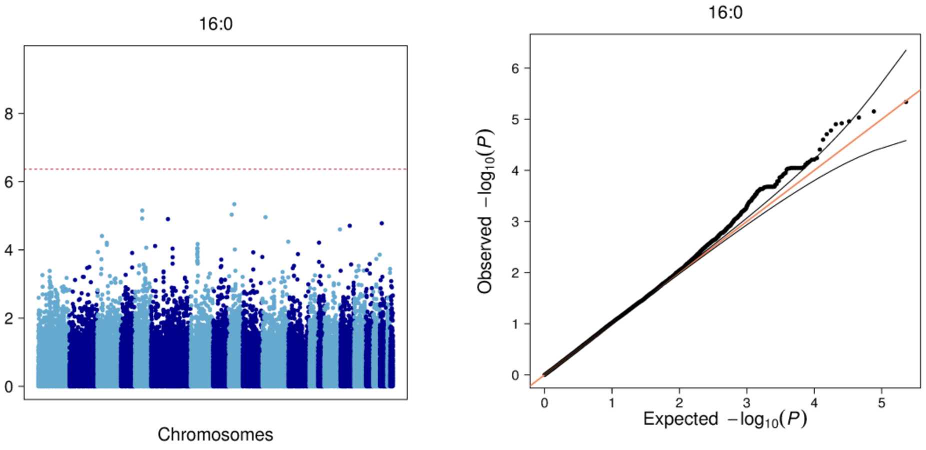
16:0
−log10(P)
Chromosomes
16:0
Observed −log10(P)
Expected −log10(P)

S

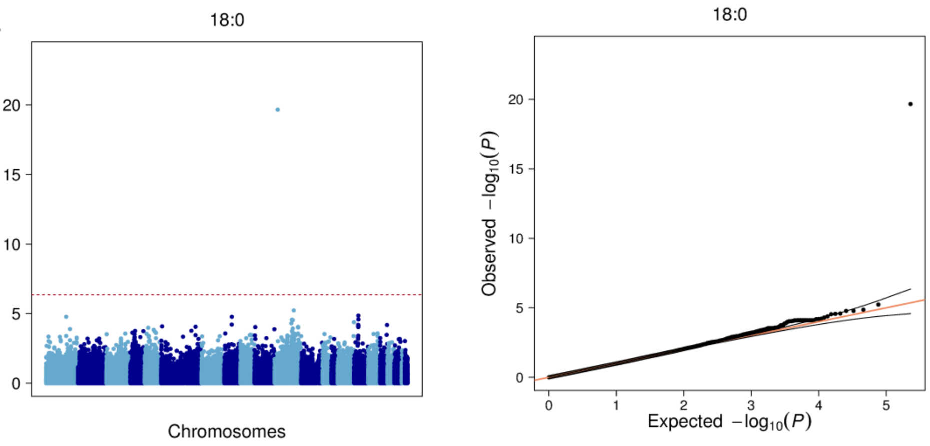
18:0
−log10(P)
Chromosomes
18:0
Observed −log10(P)
Expected −log10(P)

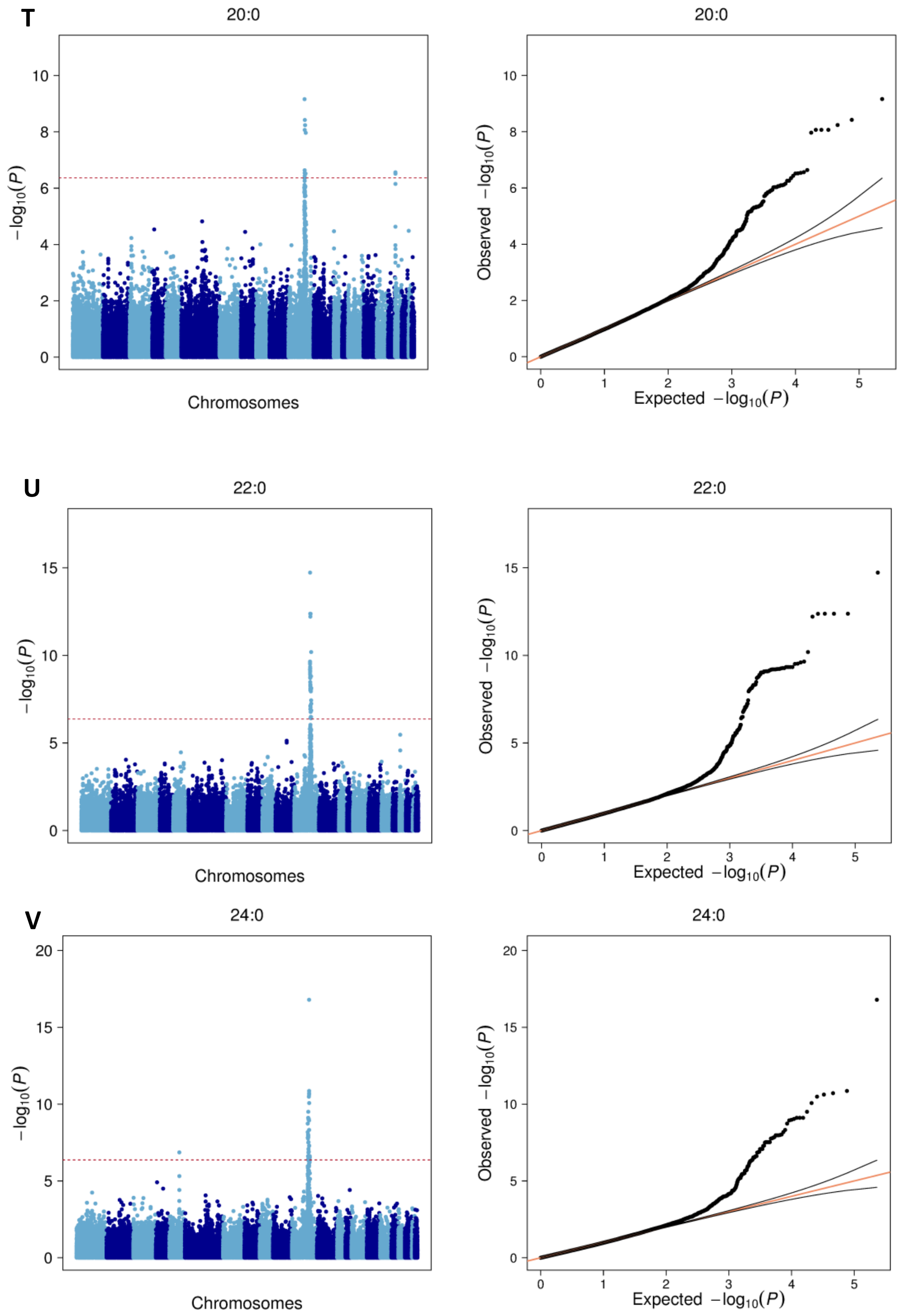
T
20:0
20:0
$-\log_{10}(P)$
Chromosomes
Observed $-\log_{10}(P)$
Expected $-\log_{10}(P)$
U
22:0
22:0
$-\log_{10}(P)$
Chromosomes
Observed $-\log_{10}(P)$
Expected $-\log_{10}(P)$
V
24:0
24:0
$-\log_{10}(P)$
Chromosomes
Observed $-\log_{10}(P)$
Expected $-\log_{10}(P)$
