# Supplementary figures and images for: Identification of Novel Genetic Determinants of Erythrocyte Membrane Fatty Acid Composition among Greenlanders
Source: PLoS Genet. 2016 Jun 24;12(6):e1006119. doi: 10.1371/journal.pgen.1006119 (PMC4920407; doi:10.1371/journal.pgen.1006119)

## S2 Fig. Flow chart of the study.

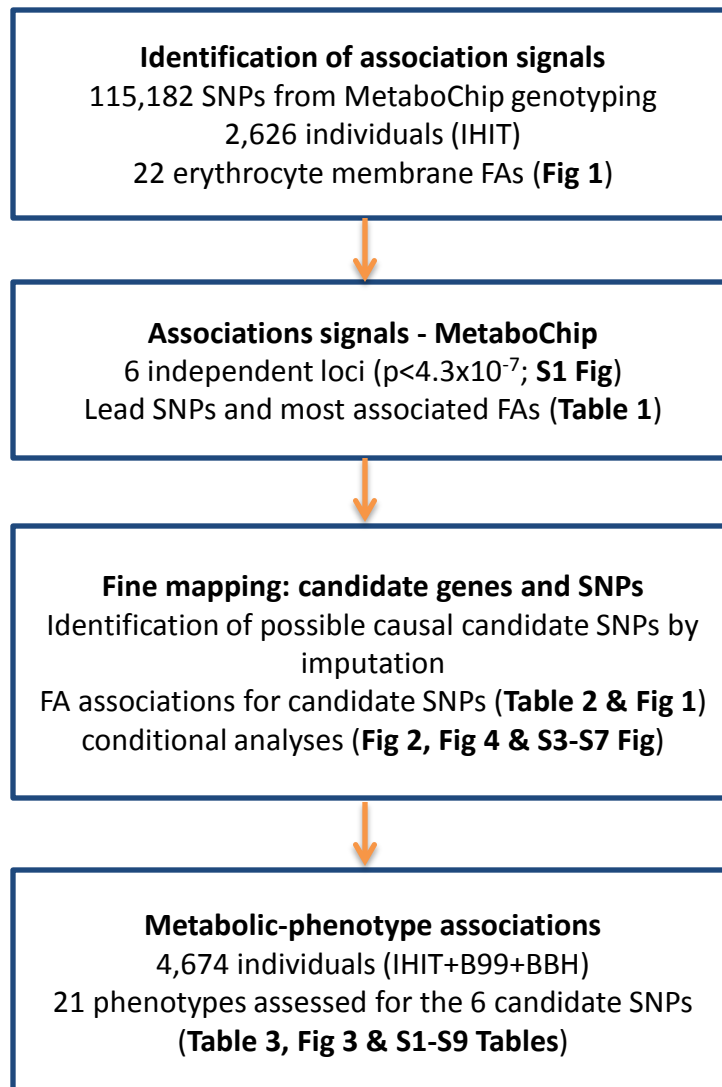

Supplement: S2 Fig — (PDF) [file pgen.1006119.s002.pdf]
