# Supplementary material for: Identification of Novel Genetic Determinants of Erythrocyte Membrane Fatty Acid Composition among Greenlanders
Source: PLoS Genet. 2016 Jun 24;12(6):e1006119. doi: 10.1371/journal.pgen.1006119 (PMC4920407; doi:10.1371/journal.pgen.1006119)

S3 Fig. Association and conditional plots. Unconditional and conditional association analyses for rs76430747 in the *ACSL6* locus with A) 11-eicosenoic acid (20:1 ω-9) and B) lignoceric acid (24:0).The association results of the unconditional analysis are colored according to the LD, which is calculated for the candidate SNP in the region. Green dots represent the results of the conditional analysis, and the circles denote the SNPs conditioned on. The p-values are based on imputation data.

A

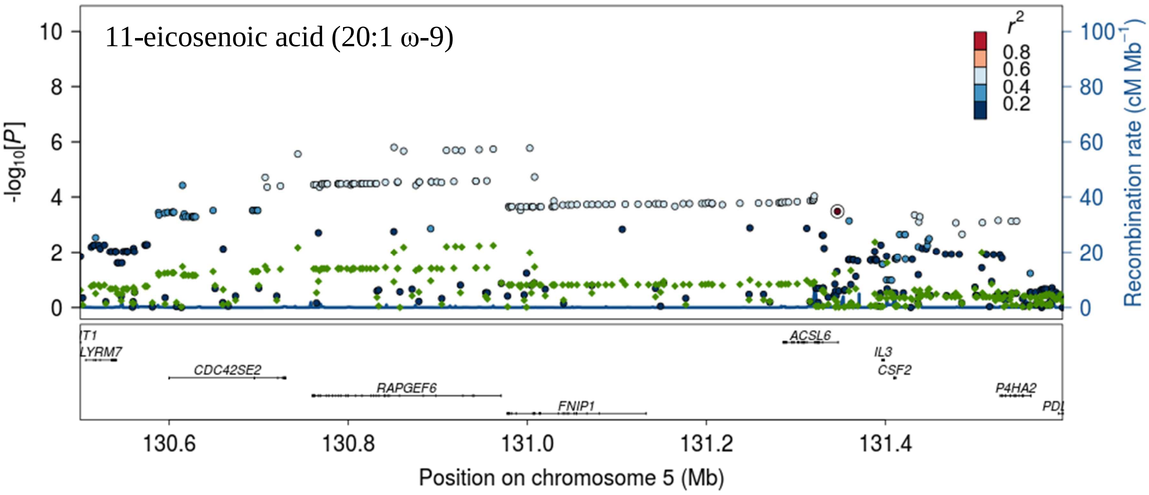

B

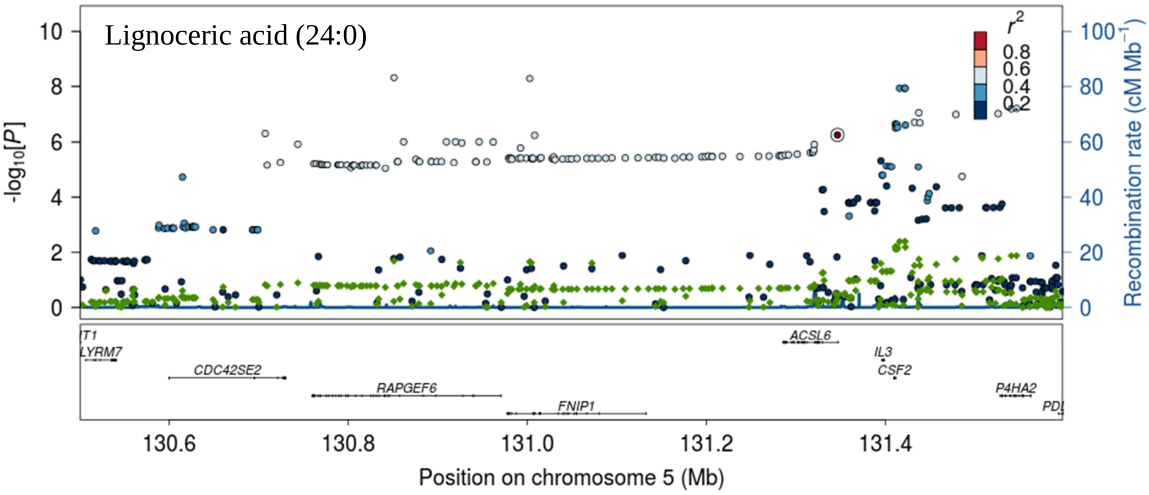

Supplement: S3 Fig — Unconditional and conditional association analyses for rs76430747 in the ACSL6 locus with A) 11-eicosenoic acid (20:1 ω-9) and B) lignoceric acid (24:0).The association results of the unconditional analysis are colored according to the LD, which is calculated for the candidate SNP in the region. Green dots represent the results of the conditional analysis, and the circles denote the SNPs conditioned on. The p-values are based on imputation data. (PDF) [file pgen.1006119.s003.pdf]
