# Supplementary material for: Identification of Novel Genetic Determinants of Erythrocyte Membrane Fatty Acid Composition among Greenlanders
Source: PLoS Genet. 2016 Jun 24;12(6):e1006119. doi: 10.1371/journal.pgen.1006119 (PMC4920407; doi:10.1371/journal.pgen.1006119)

S4 Fig. Association and conditional plots. Unconditional and conditional association analyses for rs174570 and rs80356779 in the *FADS2* and *CPT1A* loci, respectively, with A) palmitoleic acid (16:1 ω-7), B) stearic acid (18:0), C) oleic acid (18:1 ω-9), D) linoleic acid (*cis-cis*-18:2 ω-6), E) gamma-linolenic acid (18:3 ω-6), F) arachidic acid (20:0), G) 11-eicosenoic acid (20:1 ω-9), H) dihomo-gamma-linolenic acid (20:3 ω-6), I) arachidonic acid (20:4 ω-6), J) behenic acid (22:0), K) erucic acid (22:1 ω-9), L) adrenic acid (22:4 ω-6), M) docosapentaenoic acid (22:5 ω-3), N) lignoceric acid (24:0), and O) nervonic acid (24:1 ω-9).The association results of the unconditional analysis are colored according to the LD, which is calculated for the candidate SNP in the region. Green dots represent the results of the conditional analysis, and the circles denote the SNPs conditioned on. The p-values are based on imputation data.

A

Palmitoleic acid (16:1 ω-7) conditioned on rs174570

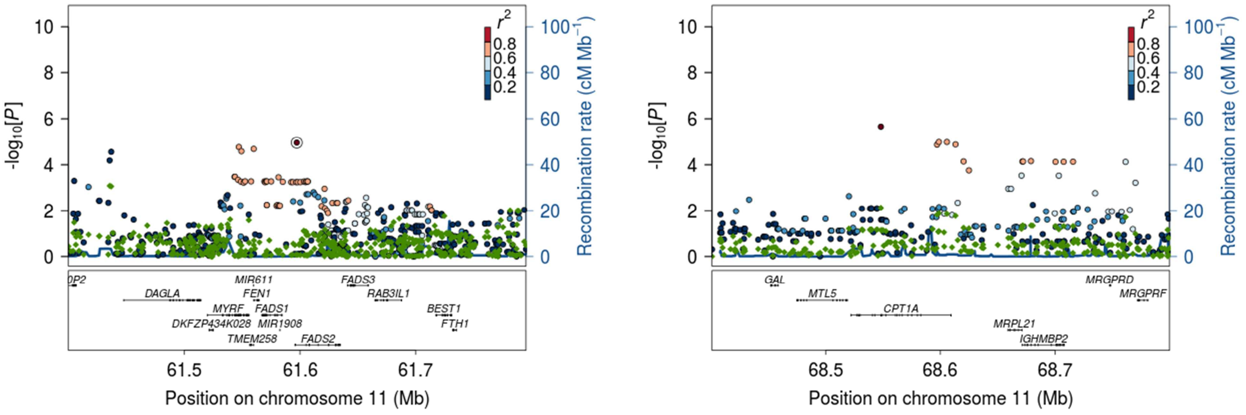

Palmitoleic acid (16:1 ω-7) conditioned on rs80356779

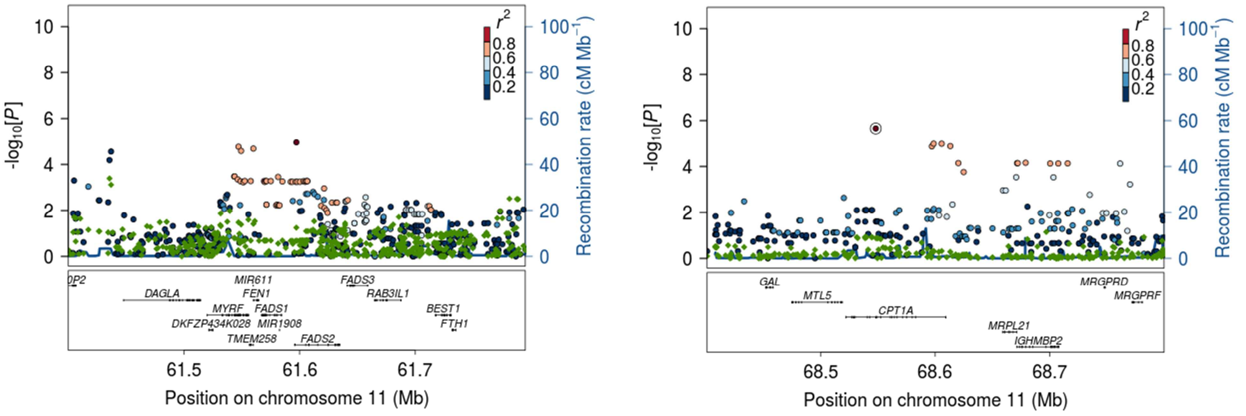

B

Stearic acid (18:0) conditioned on rs174570

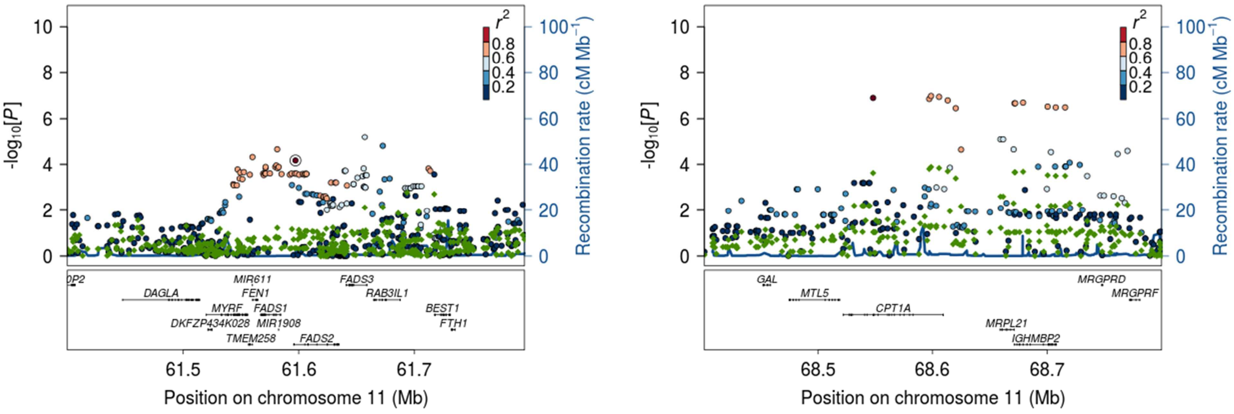

Stearic acid (18:0) conditioned on rs80356779

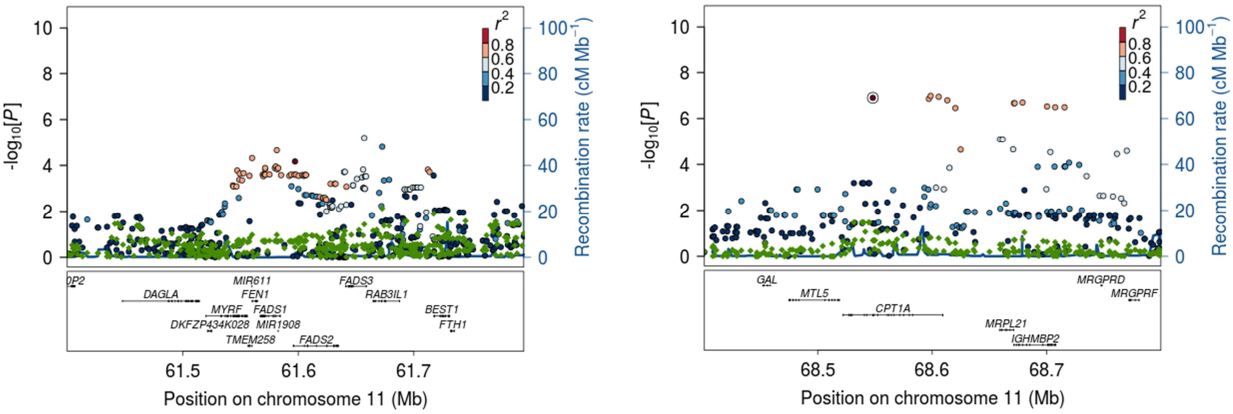

C

Oleic acid (18:1 ω-9) conditioned on rs174570

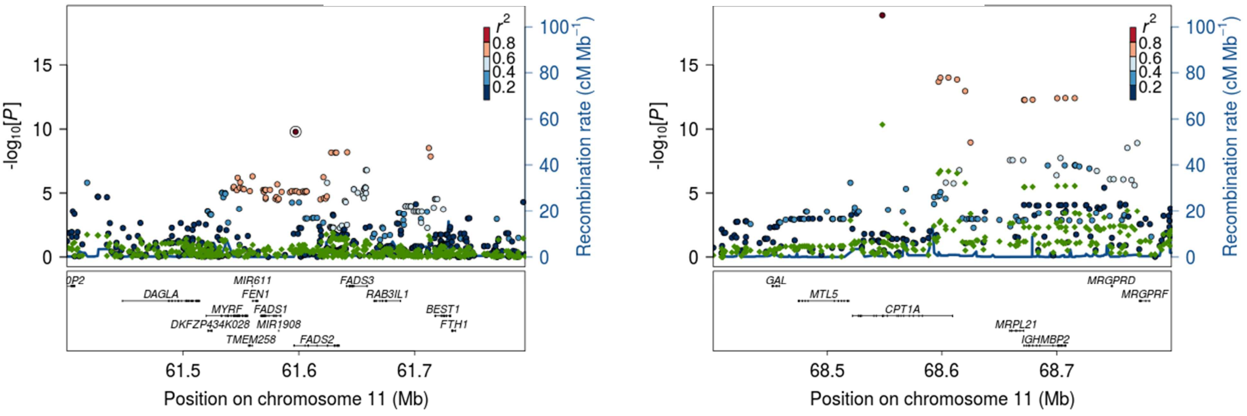

Oleic acid (18:1 ω-9) conditioned on rs80356779

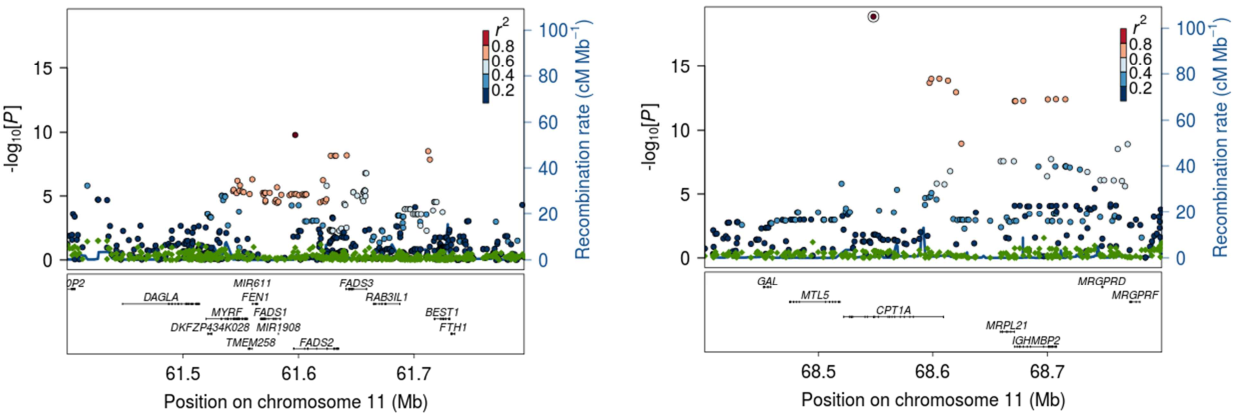

D

Linoleic acid (*cis*-*cis*-18:2 ω-6) conditioned on rs174570

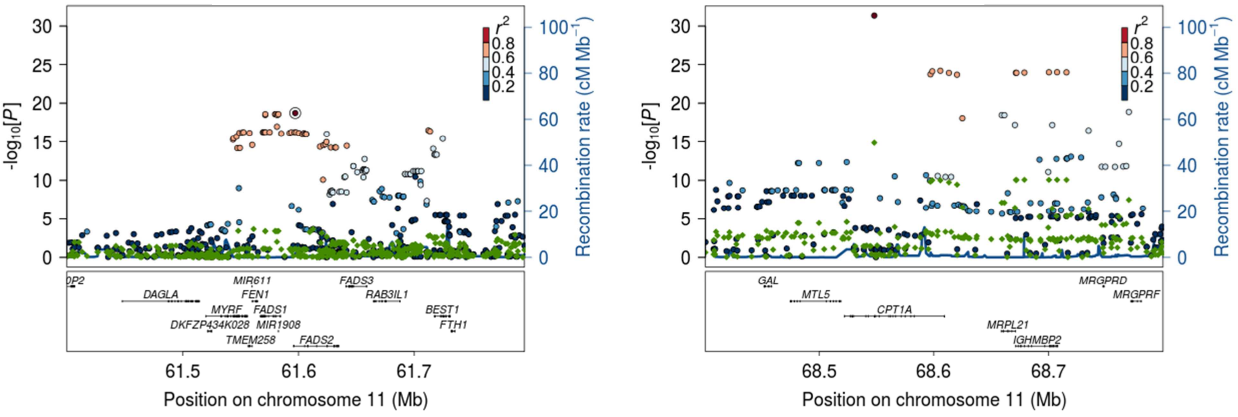

Linoleic acid (*cis*-*cis*-18:2 ω-6) conditioned on rs80356779

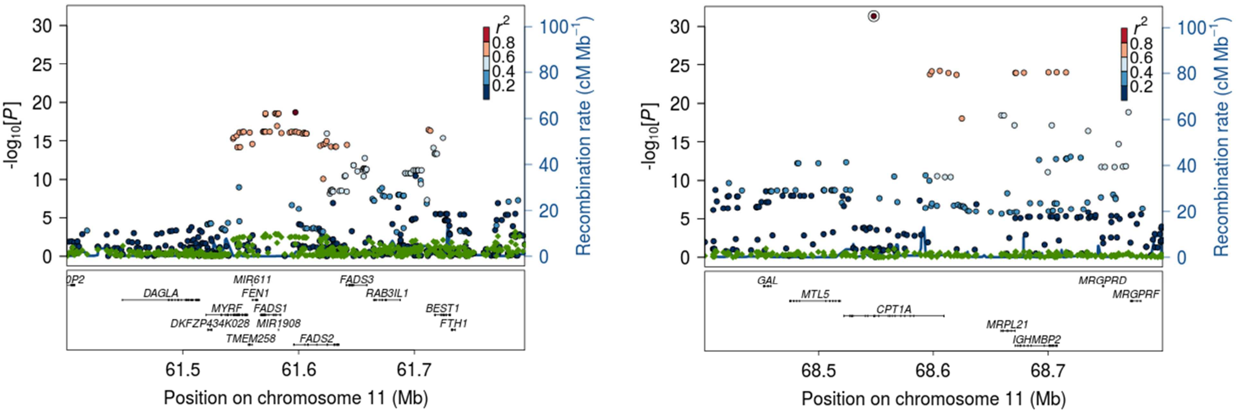

E Gamma-linolenic acid (18:3 ω-6) conditioned on rs174570

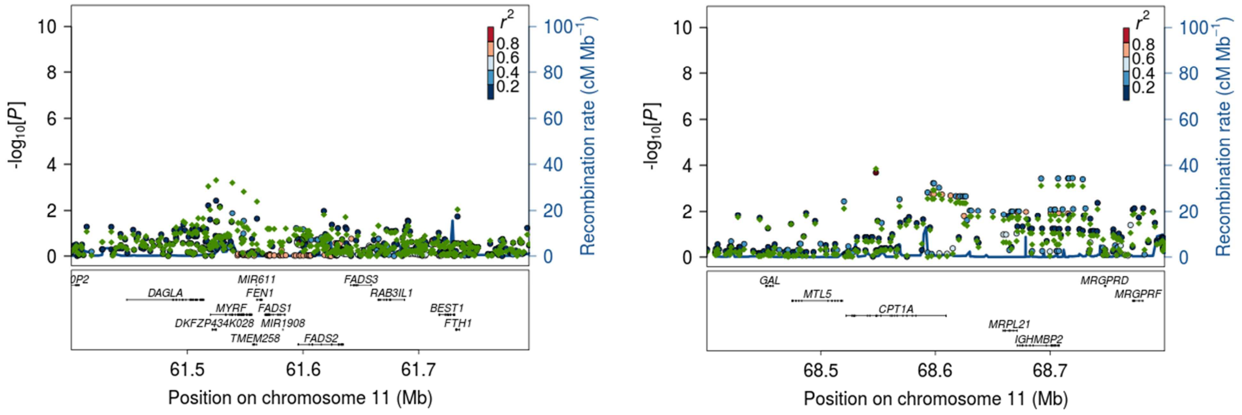

Gamma-linolenic acid (18:3 ω-6) conditioned on rs80356779

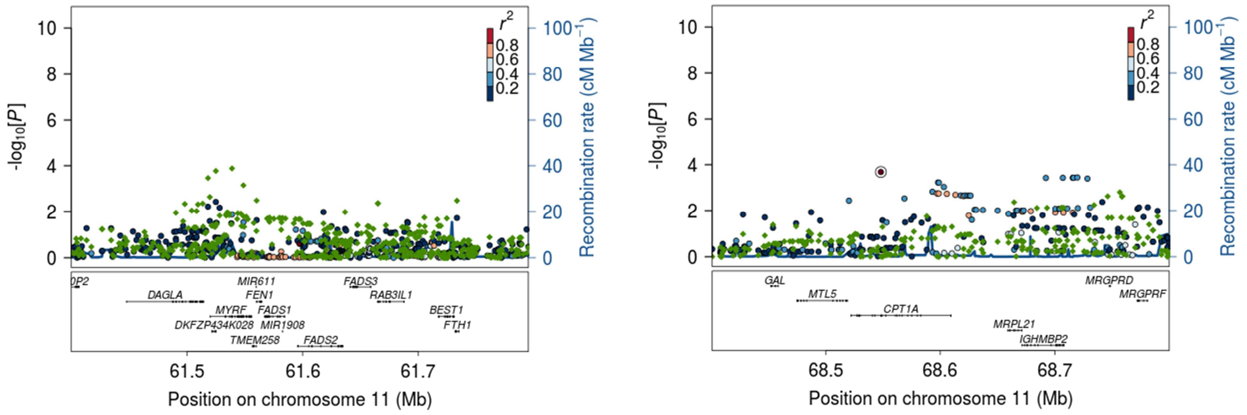

F Arachidic acid (20:0) conditioned on rs174570

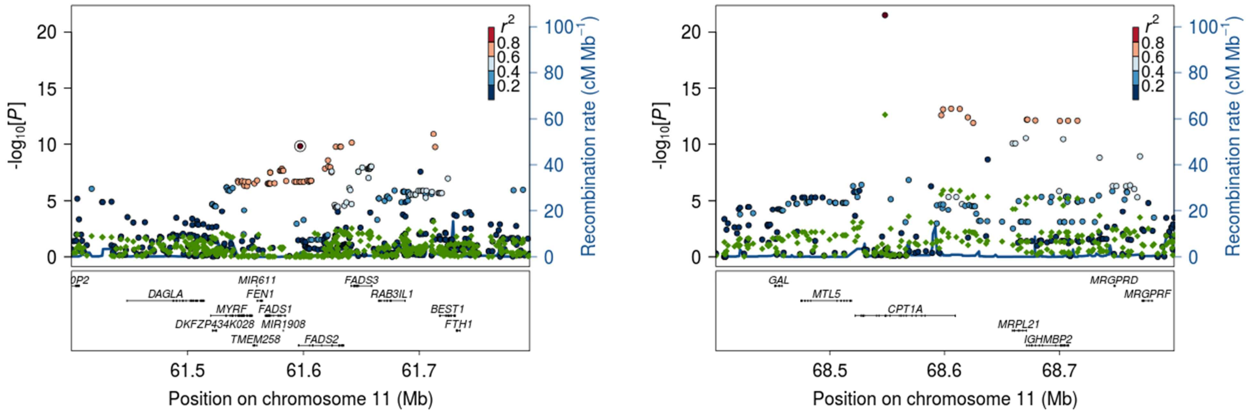

Arachidic acid (20:0) conditioned on rs80356779

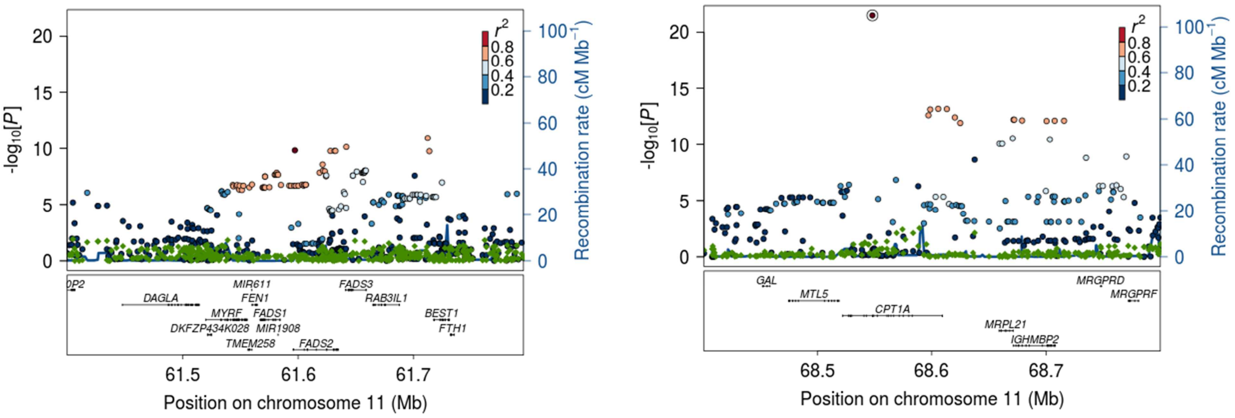

G

11-eicosenoic acid (20:1 ω-9) conditioned on rs174570

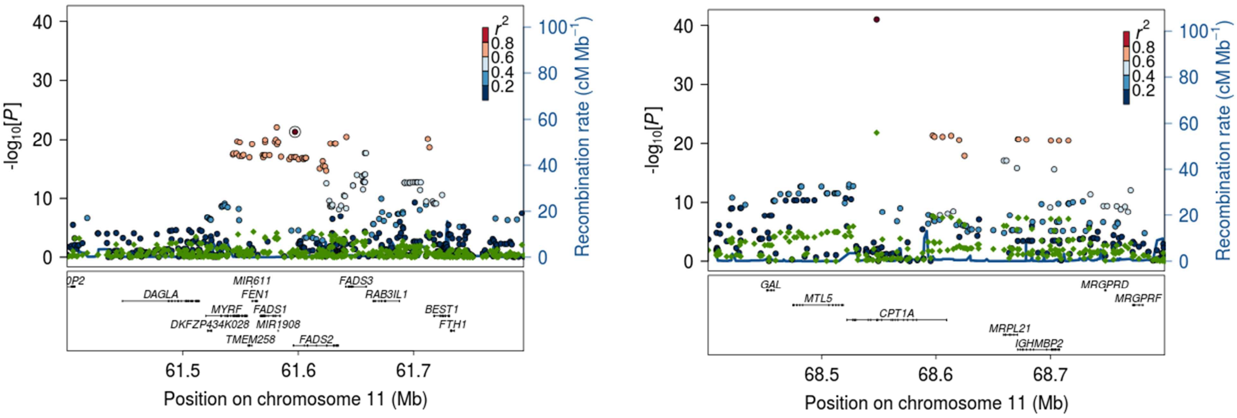

11-eicosenoic acid (20:1 ω-9) conditioned on rs80356779

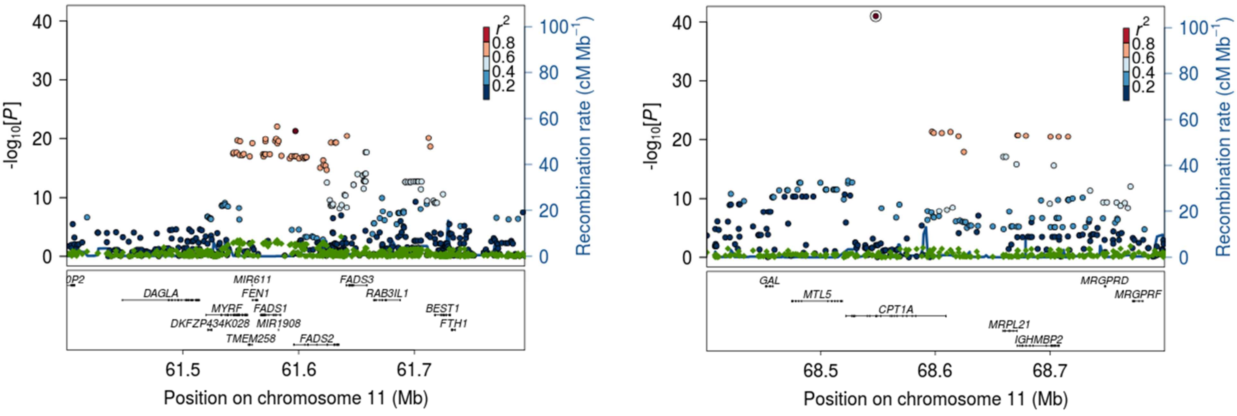

H

Dihomo-gamma-linolenic acid (20:3 ω-6) conditioned on rs174570

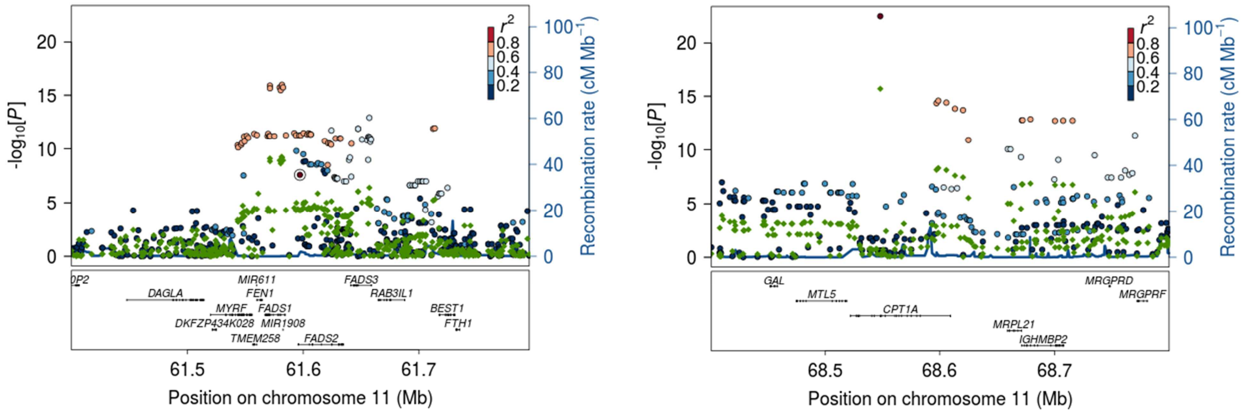

Dihomo-gamma-linolenic acid (20:3 ω-6) conditioned on rs80356779

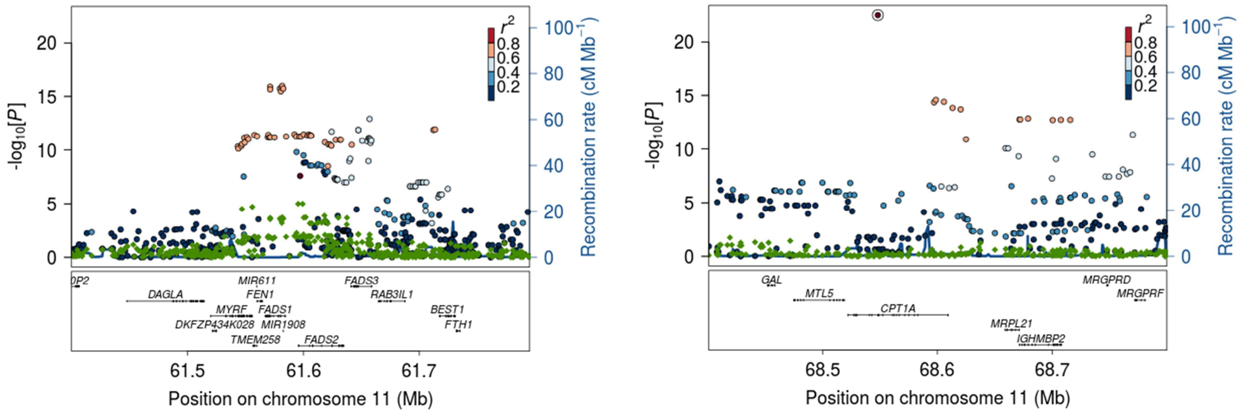

I

Arachidonic acid (20:4 ω-6) conditioned on rs174570

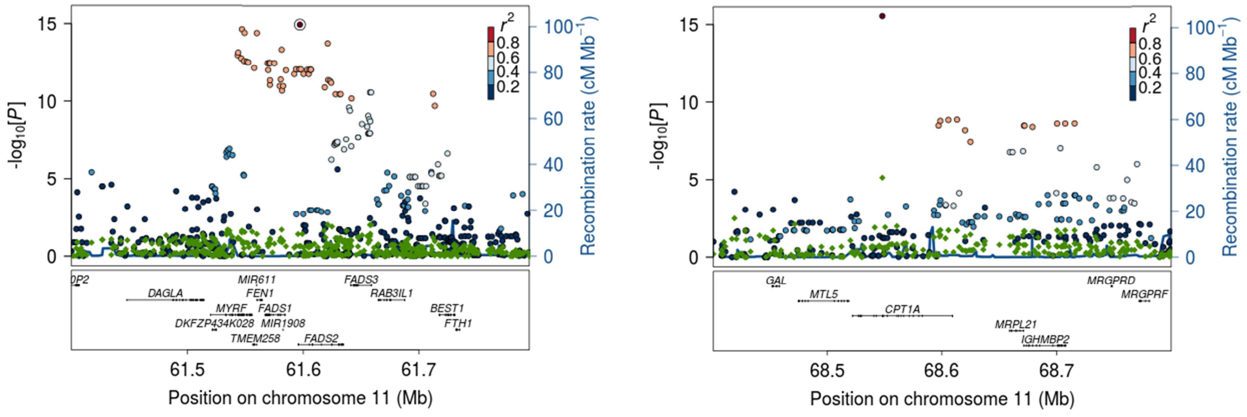

Arachidonic acid (20:4 ω-6) conditioned on rs80356779

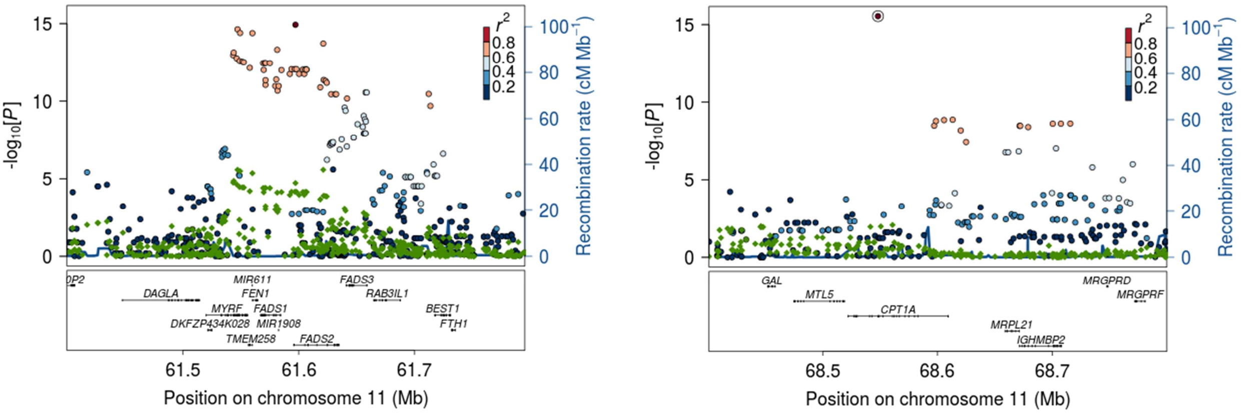

J Behenic acid (22:0) conditioned on rs174570

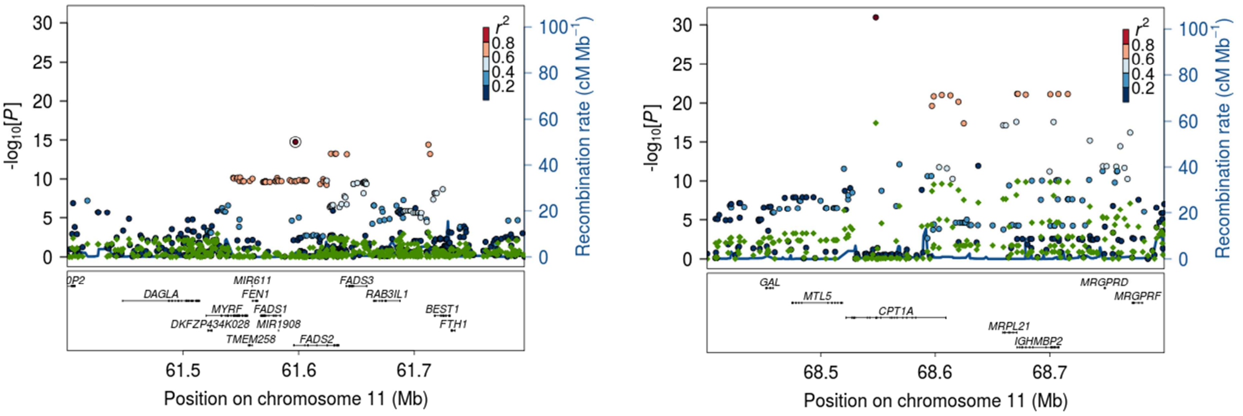

Behenic acid (22:0) conditioned on rs80356779

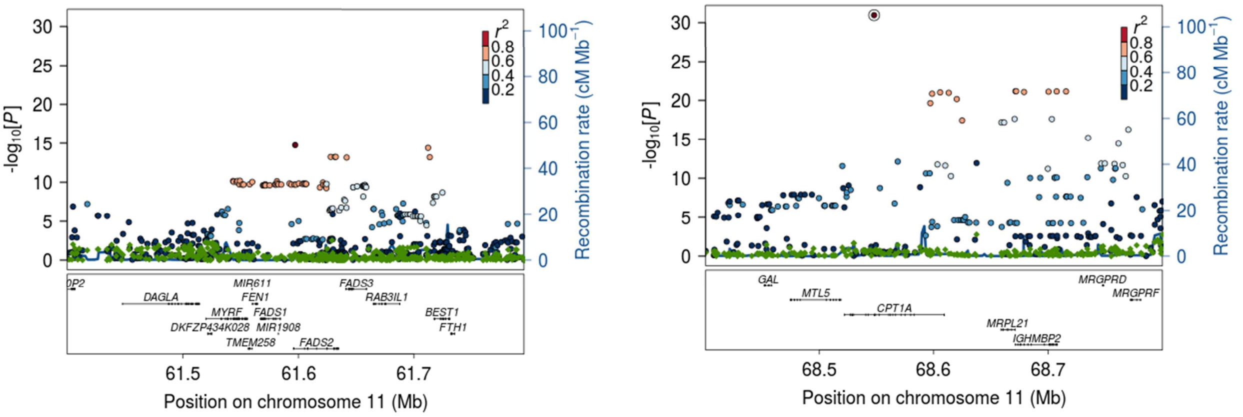

K

Erucic acid (22:1 ω-9) conditioned on rs174570

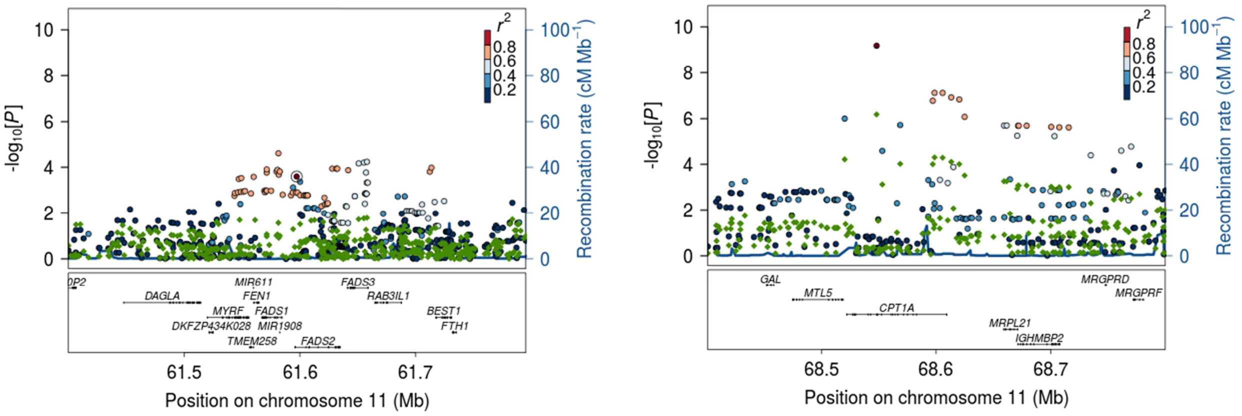

Erucic acid (22:1 ω-9) conditioned on rs80356779

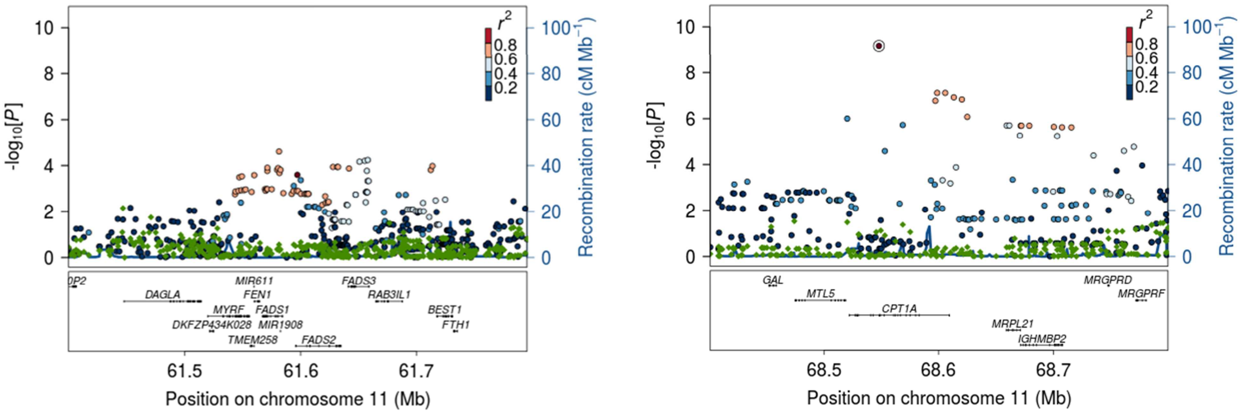

L

Adrenic acid (22:4 ω-6) conditioned on rs174570

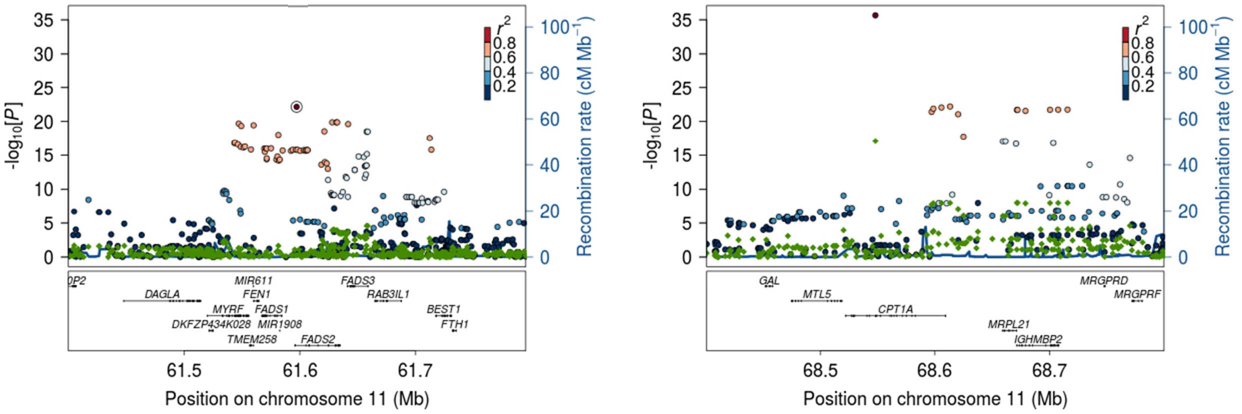

Adrenic acid (22:4 ω-6) conditioned on rs80356779

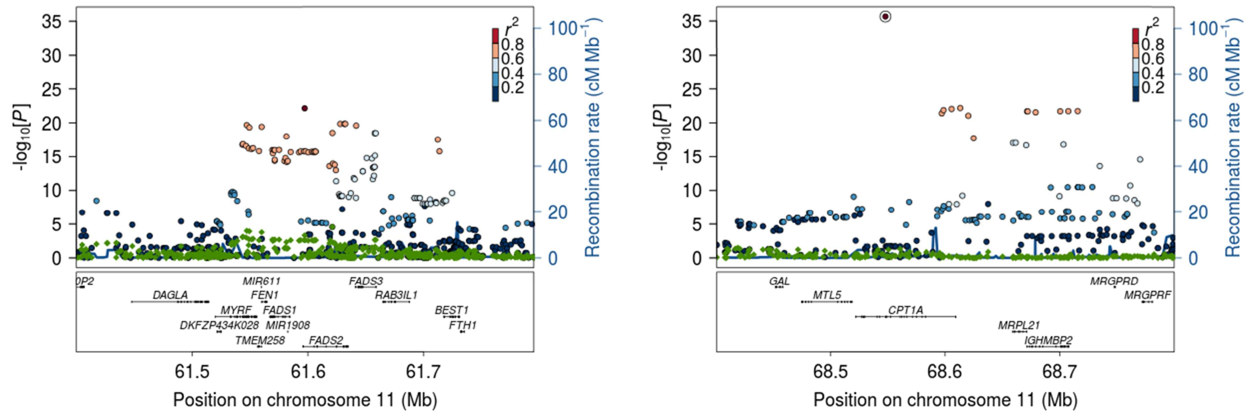

M

Docosapentaenoic acid (22:5 ω-3) conditioned on rs174570

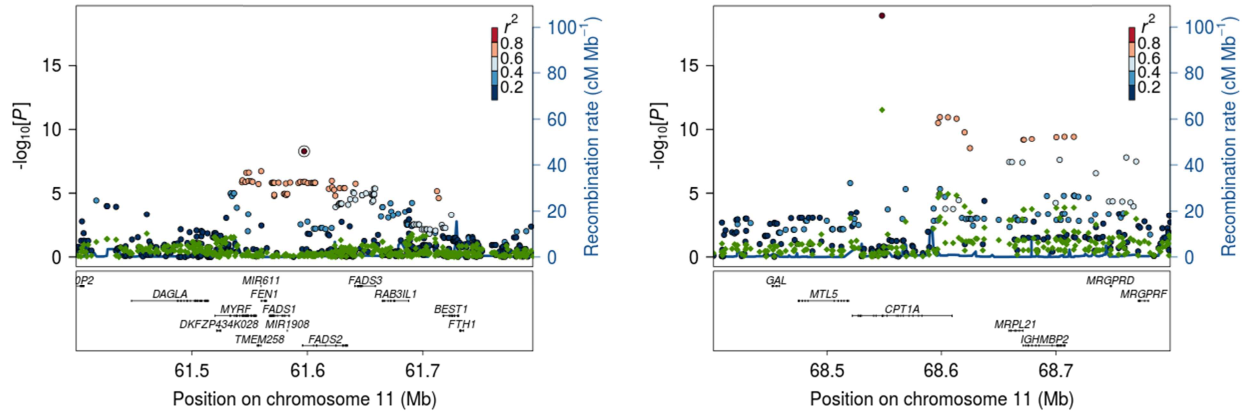

Docosapentaenoic acid (22:5 ω-3) conditioned on rs80356779

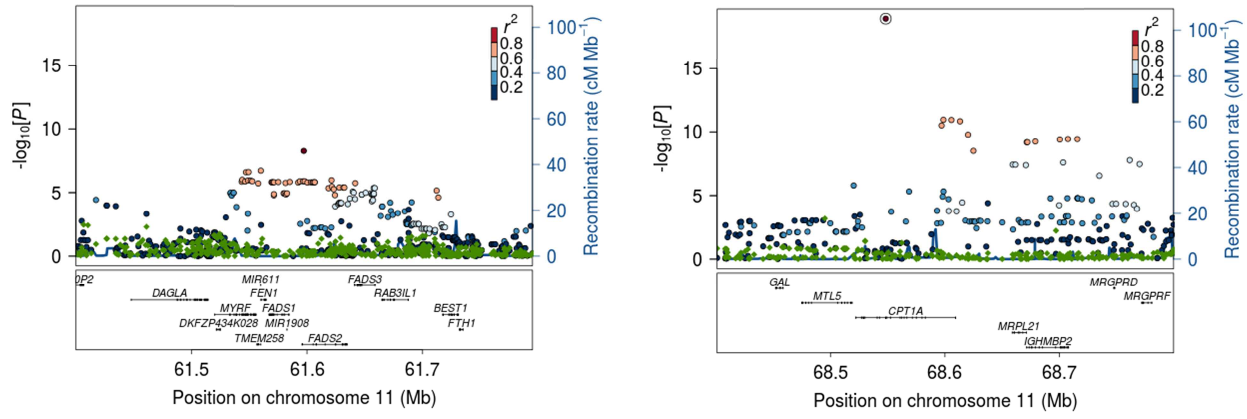

N Lignoceric acid (24:0) conditioned on rs174570

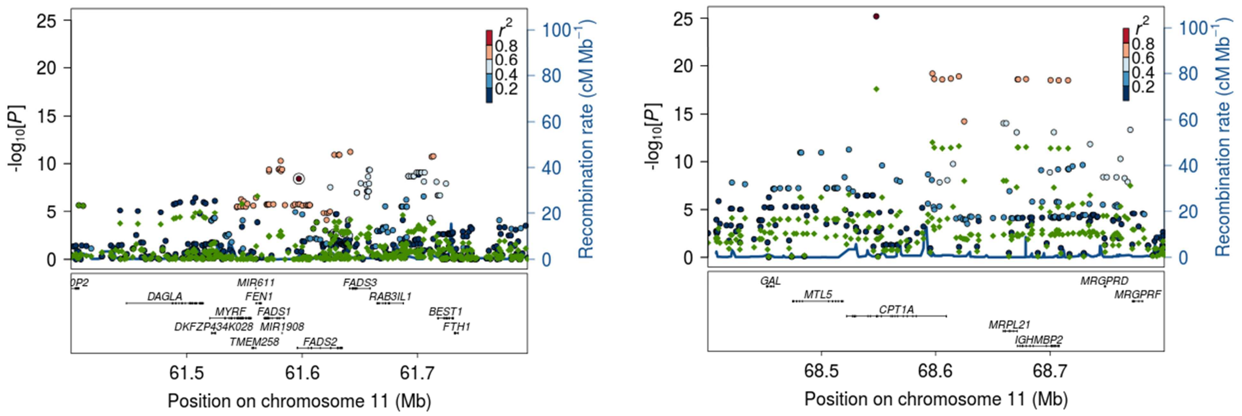

Lignoceric acid (24:0) conditioned on rs80356779

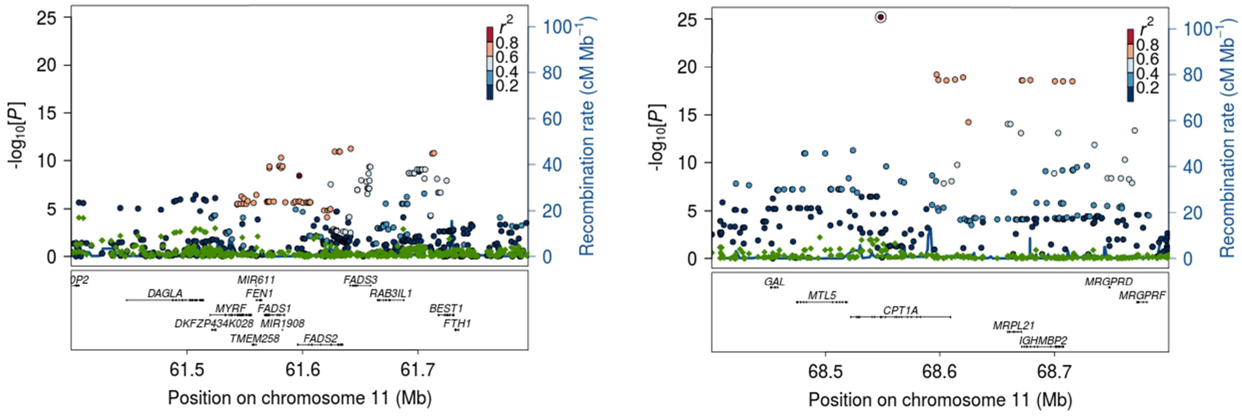

O Nervonic acid (24:1 ω-9) conditioned on rs174570

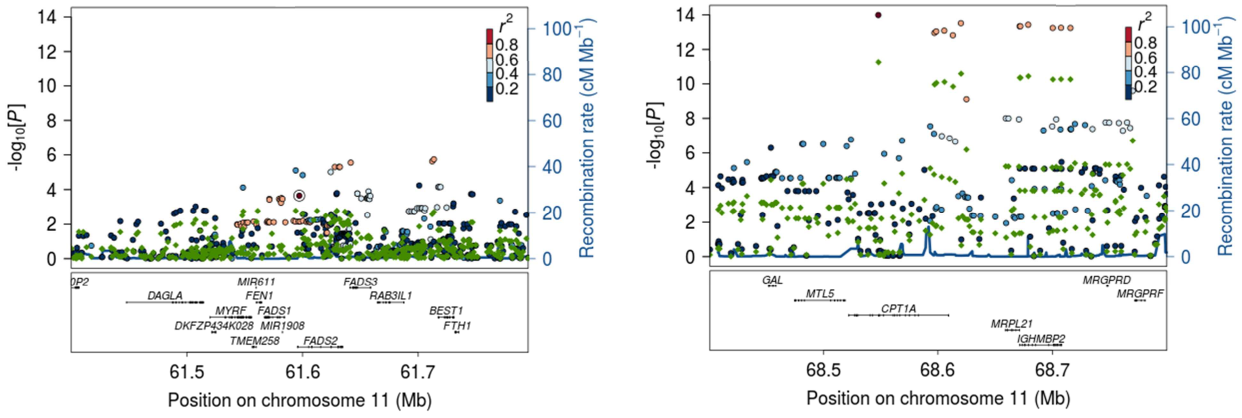

Nervonic acid (24:1 ω-9) conditioned on rs80356779

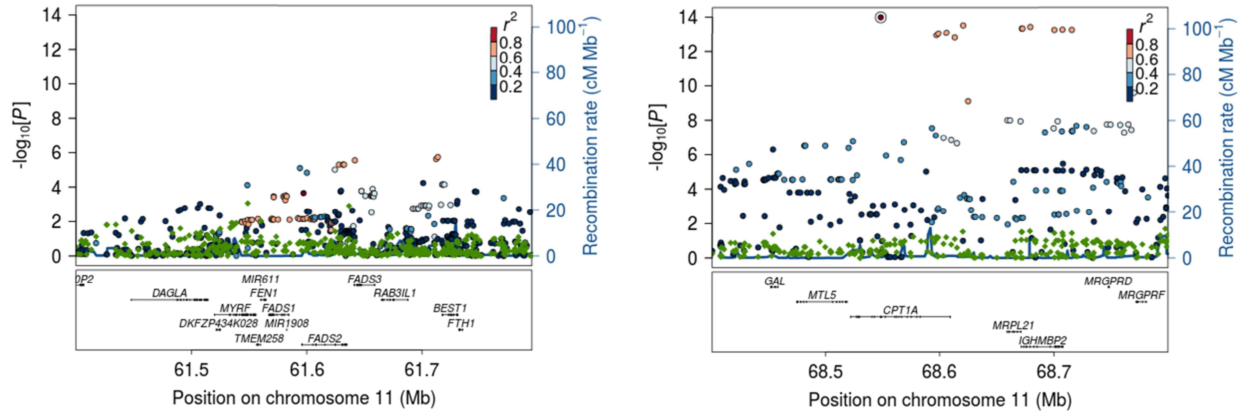

Supplement: S4 Fig — Unconditional and conditional association analyses for rs174570 and rs80356779 in the FADS2 and CPT1A loci, respectively, with A) palmitoleic acid (16:1 ω-7), B) stearic acid (18:0), C) oleic acid (18:1 ω-9), D) linoleic acid (cis-cis-18:2 ω-6), E) gamma-linolenic acid (18:3 ω-6), F) arachidic acid (20:0), G) 11-eicosenoic acid (20:1 ω-9), H) dihomo-gamma-linolenic acid (20:3 ω-6), I) arachidonic acid (20:4 ω-6), J) behenic acid (22:0), K) erucic acid (22:1 ω-9), L) adrenic acid (22:4 ω-6), M) docosapentaenoic acid (22:5 ω-3), N) lignoceric acid (24:0), and O) nervonic acid (24:1 ω-9).The association results of the unconditional analysis are colored according to the LD, which is calculated for the candidate SNP in the region. Green dots represent the results of the conditional analysis, and the circles denote the SNPs conditioned on. The p-values are based on imputation data. (PDF) [file pgen.1006119.s004.pdf]
