# Supplementary material for: Identification of Novel Genetic Determinants of Erythrocyte Membrane Fatty Acid Composition among Greenlanders
Source: PLoS Genet. 2016 Jun 24;12(6):e1006119. doi: 10.1371/journal.pgen.1006119 (PMC4920407; doi:10.1371/journal.pgen.1006119)
Supplement: S7 Fig — Unconditional and conditional association analyses for rs6035106 in the DTD1 locus with A) eicosapentaenoic acid (20:5 ω-3), B) docosapentaenoic acid (22:5 ω-3), C) docosahexaenoic acid (22:6 ω-3), D) dihomo-gamma-linolenic acid (20:3 ω-6), E) arachidonic acid (20:4 ω-6), F) adrenic acid (22:4 ω-6), G) oleic acid (18:1 ω-9), H) palmitic acid (16:0), and I) behenic acid (22:0).The association results of the unconditional analysis are colored according to the LD, which is calculated for the candidate SNP in the region. Green dots represent the results of the conditional analysis, and the circles denote the SNPs conditioned on. The p-values are based on imputation data. (PDF) [file pgen.1006119.s007.pdf]

S7 Fig. Association and conditional plots. Unconditional and conditional association analyses for rs6035106 in the *DTD1* locus with A) eicosapentaenoic acid (20:5 ω-3), B) docosapentaenoic acid (22:5 ω-3), C) docosahexaenoic acid (22:6 ω-3), D) dihomo-gamma-linolenic acid (20:3 ω-6), E) arachidonic acid (20:4 ω-6), F) adrenic acid (22:4 ω-6), G) oleic acid (18:1 ω-9), H) palmitic acid (16:0), and I) behenic acid (22:0).The association results of the unconditional analysis are colored according to the LD, which is calculated for the candidate SNP in the region. Green dots represent the results of the conditional analysis, and the circles denote the SNPs conditioned on. The p-values are based on imputation data.

A

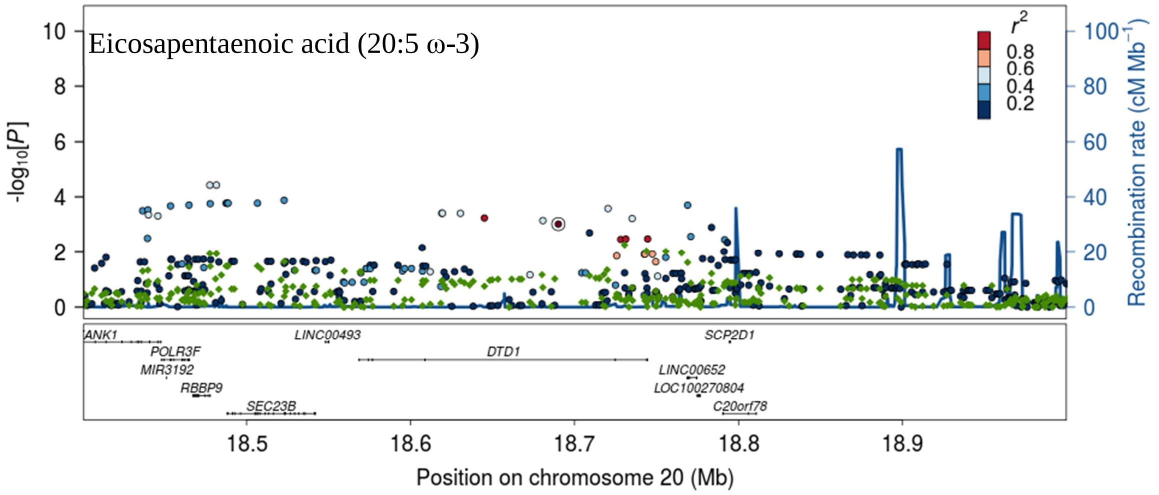

B

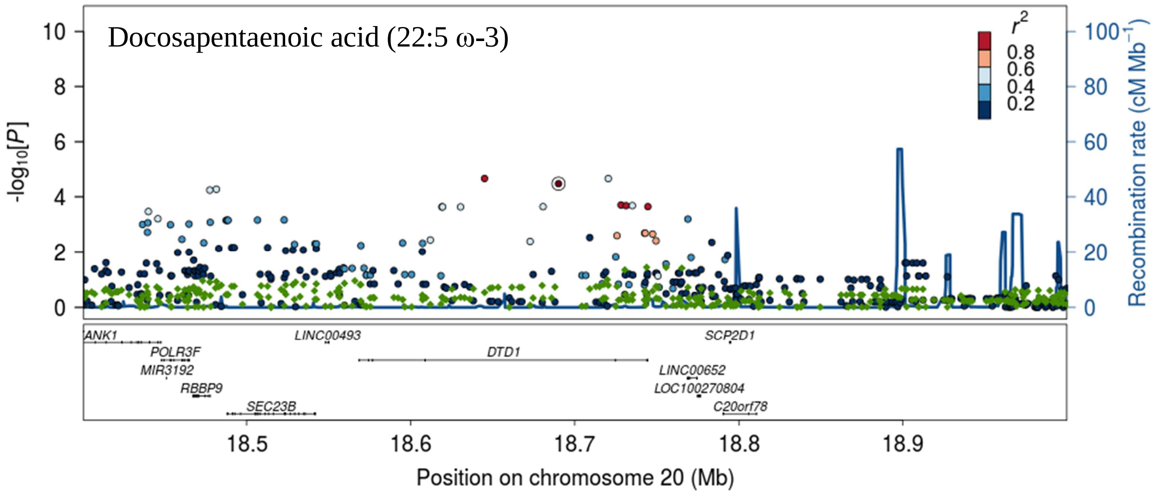

C

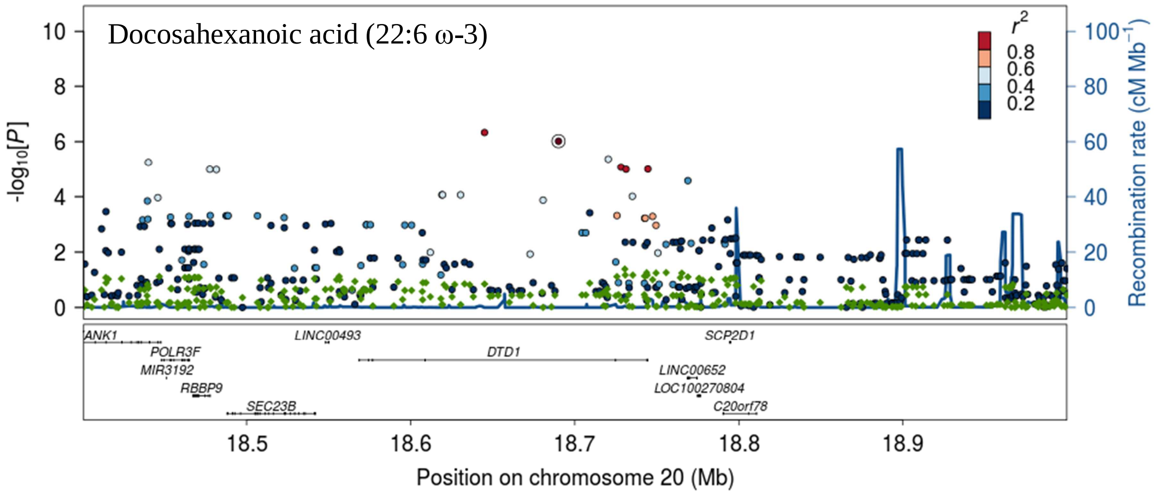
Docosahexanoic acid (22:6 ω-3)
-log10[P]
Recombination rate (cM Mb−1)
r2
0.8
0.6
0.4
0.2
ANK1
POLR3F
MIR3192
RBBP9
SEC23B
LINC00493
DTD1
SCP2D1
LINC00652
LOC100270804
C20orf78
18.5
18.6
18.7
18.8
18.9
Position on chromosome 20 (Mb)

D

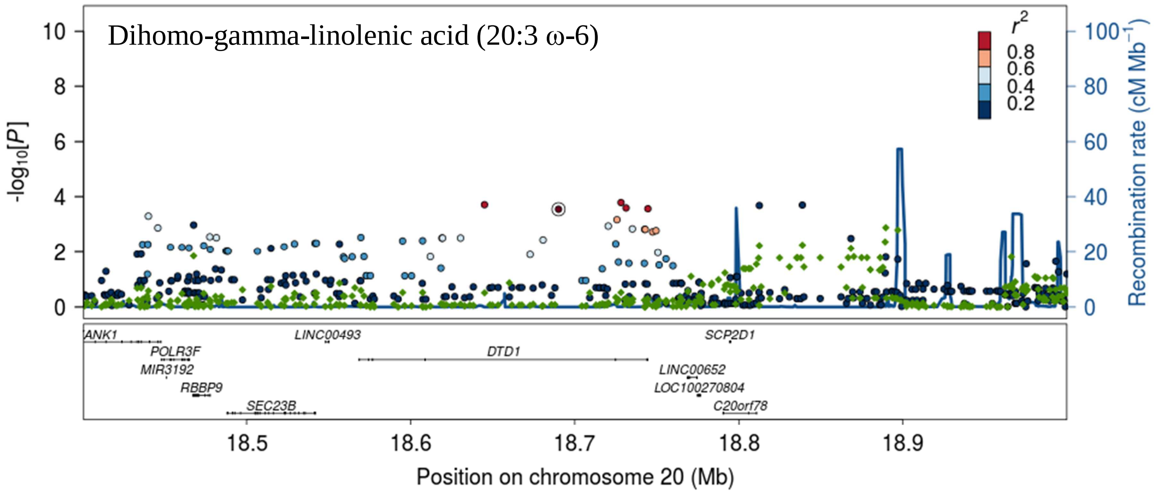
Dihomo-gamma-linolenic acid (20:3 ω-6)
-log10[P]
Recombination rate (cM Mb−1)
r2
0.8
0.6
0.4
0.2
ANK1
POLR3F
MIR3192
RBBP9
SEC23B
LINC00493
DTD1
SCP2D1
LINC00652
LOC100270804
C20orf78
18.5
18.6
18.7
18.8
18.9
Position on chromosome 20 (Mb)

E

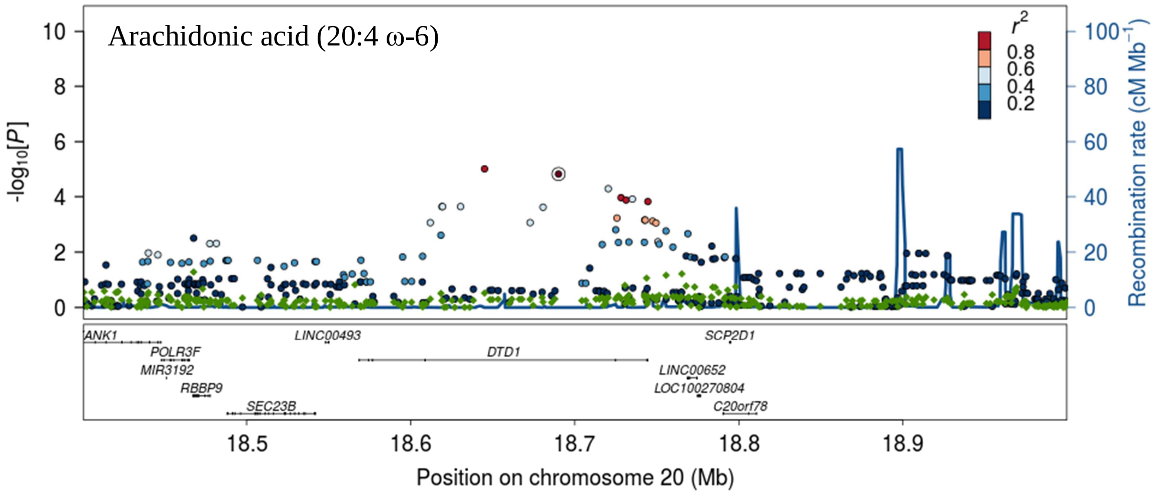
Arachidonic acid (20:4 ω-6)
-log10[P]
r2
0.8
0.6
0.4
0.2
Recombination rate (cM Mb−1)
ANK1
POLR3F
MIR3192
RBBP9
SEC23B
LINC00493
DTD1
SCP2D1
LINC00652
LOC100270804
C20orf78
18.5
18.6
18.7
18.8
18.9
Position on chromosome 20 (Mb)

F

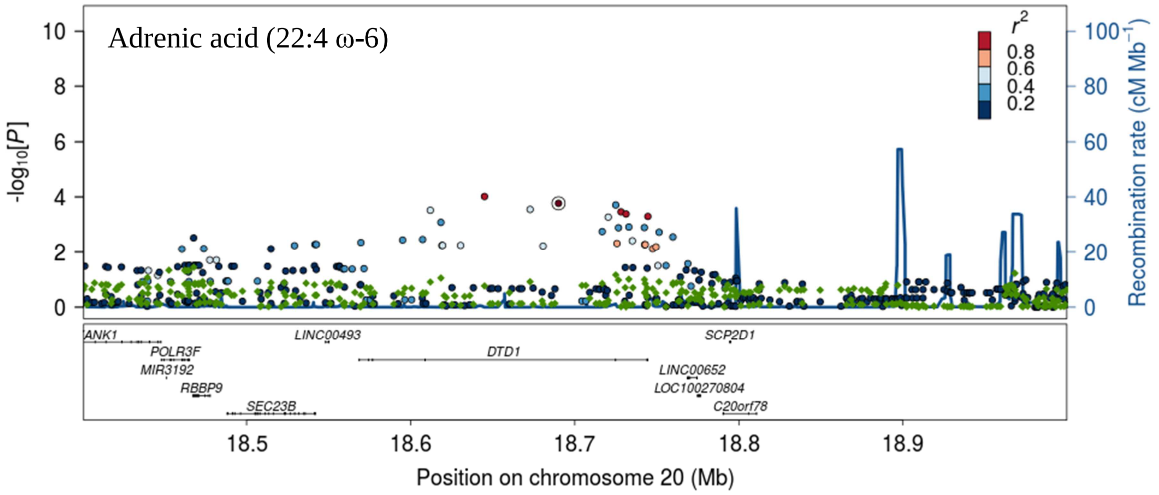
Adrenic acid (22:4 ω-6)
-log10[P]
r2
0.8
0.6
0.4
0.2
Recombination rate (cM Mb−1)
ANK1
POLR3F
MIR3192
RBBP9
SEC23B
LINC00493
DTD1
SCP2D1
LINC00652
LOC100270804
C20orf78
18.5
18.6
18.7
18.8
18.9
Position on chromosome 20 (Mb)

G

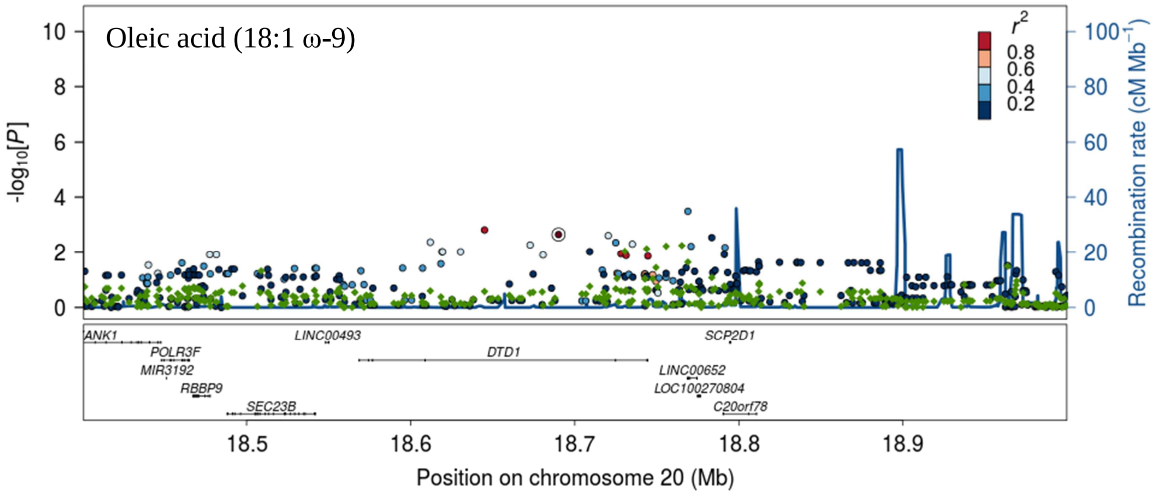
Oleic acid (18:1 ω-9)
r^2
0.8
0.6
0.4
0.2
-log10[P]
Recombination rate (cM Mb^{-1})
ANK1
POLR3F
MIR3192
RBBP9
SEC23B
LINC00493
DTD1
SCP2D1
LINC00652
LOC100270804
C20orf78
18.5
18.6
18.7
18.8
18.9
Position on chromosome 20 (Mb)

H

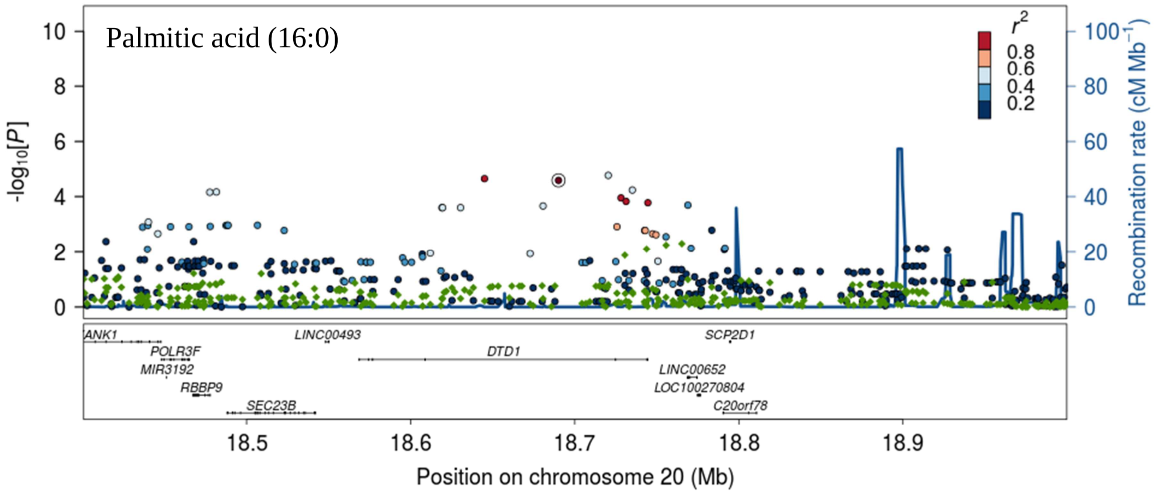
Palmitic acid (16:0)
r^2
0.8
0.6
0.4
0.2
-log10[P]
Recombination rate (cM Mb^{-1})
ANK1
POLR3F
MIR3192
RBBP9
SEC23B
LINC00493
DTD1
SCP2D1
LINC00652
LOC100270804
C20orf78
18.5
18.6
18.7
18.8
18.9
Position on chromosome 20 (Mb)

I

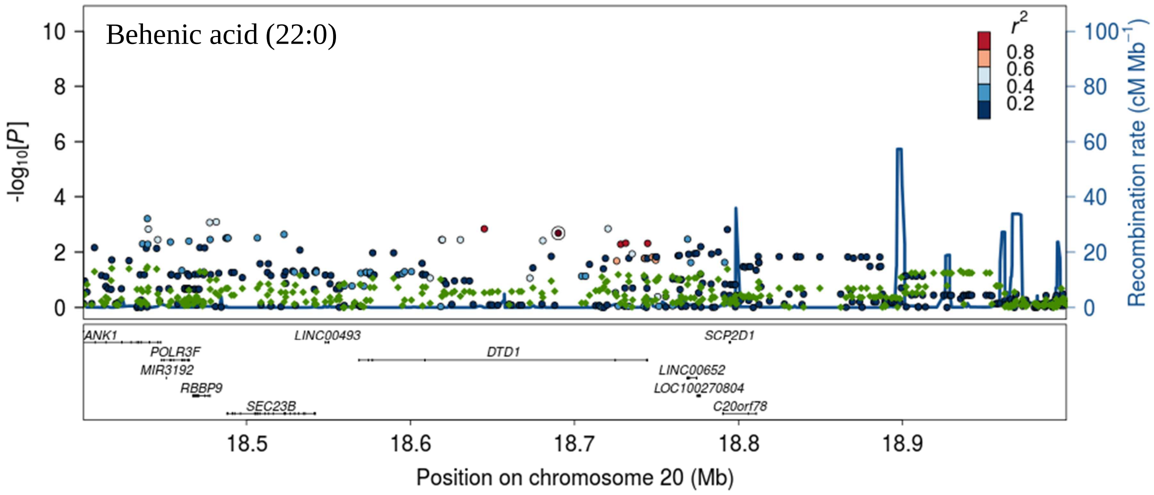

Behenic acid (22:0)
-log10[P]
Recombination rate (cM Mb−1)
r2
0.8
0.6
0.4
0.2
ANK1
POLR3F
MIR3192
RBBP9
SEC23B
LINC00493
DTD1
SCP2D1
LINC00652
LOC100270804
C20orf78
18.5
18.6
18.7
18.8
18.9
Position on chromosome 20 (Mb)
